# Web-based Cellpose‑SAM segmentation enables quantification of cross-sectional area in formalin-fixed paraffin-embedded skeletal muscle sections stained with hematoxylin and eosin

**DOI:** 10.1371/journal.pone.0358476

**Published:** 2026-09-21

**Authors:** Jin-Hyang Park, Hye-Jin Yoon, Soo-Ji Kim, Jae-Ryong Kim

**Affiliations:** Department of Biochemistry and Molecular Biology, College of Medicine Yeungnam University, Daegu, Republic of Korea; Lorestan University, IRAN, ISLAMIC REPUBLIC OF

## Abstract

Cross‑sectional area (CSA) of skeletal myofibers is a primary morphometric indicator of atrophy and regeneration, yet it is most commonly quantified on cryosections after immunofluorescent labeling of basal lamina proteins, which requires specialized reagents and equipment and limits reuse of routine histology slides. Here we present a practical, fully web‑based workflow that leverages Cellpose‑SAM on a public browser interface to segment myofibers directly from formalin-fixed paraffin-embedded (FFPE) skeletal muscle sections stained with hematoxylin and eosin (H&E) or toluidine blue (TB), followed by downstream region‑of‑interest (ROI) curation and batch morphometry in Fiji/ImageJ. We validated this approach across whole muscles and tissue preparations in mice. In exemplar experiments on both FFPE and frozen sections of mouse soleus, CSA distributions from H&E and TB showed no significant differences. A sciatic nerve crush injury model exhibited a significant reduction in mean and median CSA in mouse soleus sections with a characteristic left-shift in distribution relative to sham controls, consistent with rapid denervation atrophy. This accessible pipeline lowers experimental barriers, enables analysis of archived H&E or TB slides, and supports reproducible, high-throughput morphometry for neuromuscular research.

## Introduction

Quantifying myofiber cross-sectional area (CSA) is fundamental in skeletal muscle biology because it directly reflects fiber size changes during atrophy, hypertrophy, and regeneration [1,2]. The prevailing workflow relies on cryosections immuno-stained for basal lamina markers (laminin, dystrophin, or collagen IV) to delineate fiber borders, followed by semi-automated region-of-interest (ROI) extraction and area measurement [1,3]. Although well-established, fluorescence-based CSA analysis requires cryosection handling, fluorophore stability management, dedicated microscopes, and careful batch standardization [1,3]. Moreover, this approach is poorly suited for formalin-fixed paraffin-embedded (FFPE) tissue archives, which are routinely produced in both research and clinical settings [4,5]. These limitations have prevented widespread adoption of quantitative CSA analysis, particularly in clinical and translational settings where FFPE archives predominate.

Recent semi-automated toolchains such as MuscleJ [6], Open-CSAM [7], and related protocols [8] provide computational assistance but still require immunolabels and specialized imaging hardware, limiting scalability and multi-site reproducibility. In contrast, H&E staining remains ubiquitous in histopathology and is compatible with both frozen and FFPE tissues, opening the possibility of retrospective analysis if CSA segmentation can be performed directly on bright-field images [4,5].

Generalist deep learning methods have substantially lowered technical barriers: Cellpose segments across diverse imaging modalities [9], and Cellpose 2.0 adds human-in-the-loop fine-tuning for rapid domain adaptation [10]. Meta's Segment Anything Model (SAM) provides zero-shot, promptable segmentation masks [11]. The recent integration of SAM within Cellpose-SAM, exposed via a browser-based Hugging Face interface, eliminates local GPU and Python installation requirements, making the method accessible to investigators without specialized computational infrastructure.

We hypothesized that combining a web-based Cellpose-SAM (CS) segmentation with standardized morphological filtering would enable accurate CSA quantification from routine bright-field histology. Here, we developed and validated a web-first CSA quantification workflow that leverages Cellpose-SAM for segmentation and Fiji/ImageJ with MorphoLibJ for mask-to-ROI curation. We validated this approach across different staining methods (H&E and TB), tissue preparations (FFPE and frozen), and biological conditions (sham vs denervation injury). This approach enables standardized, installation-free myofiber morphometry on routine bright-field tissue sections from both research and archived clinical samples.

## Materials and methods

### Materials

Male C57BL/6 mice were purchased from Hyochang Science Co., Ltd. (Daegu, Republic of Korea). Avertin (2,2,2-tribromoethanol; Cat. No. T48402), paraffin (Cat. No. K95882661), toluidine blue O (Cat. No. T3260), Harris hematoxylin (Cat. No. HHS32), and eosin Y (Cat. No. E4382) were purchased from Sigma-Aldrich, Merck KGaA (St. Louis, MO, USA). Alexa Fluor 555-conjugated wheat germ agglutinin (WGA: ThermoFisher Scientific, Waltham, MA, USA; Cat. No. W32464), 10% neutral buffered formalin (BBC Biochemical, Mount Vernon, WA, USA; Cat. No. 234138), optimal cutting temperature (OCT) compound (Sakura Finetek, Torrance, CA, USA; Cat. No. 4583), Dako fluorescence mounting medium (Agilent Technologies Inc., Santa Clara, CA, USA; Cat. No. S3023), and isopentane (Samchun Chemicals, Seoul, Republic of Korea) were used in this study.

### Sciatic nerve crush injury (SCI) in mice

Animal experimental procedures were approved by the Institutional Animal Care and Use Committee of Yeungnam University College of Medicine and conducted in accordance with institutional guidelines (YUMC-AEC2024–008). The total duration of the animal experiment was 14 days, including acclimatization, surgery, and endpoint analysis. Mice were housed under standard laboratory conditions with free access to food and water. All animal procedures were performed by trained personnel who had received institutional training in animal handling, surgery, anesthesia, and euthanasia. A total of seven 11-week-old male C57BL/6 mice were randomly divided into a sham group (*n* = 3) and a sciatic nerve crush injury (SCI) group (*n* = 4) using a random number generator. SCI was induced according to the established protocols [12–14]. Mice were anesthetized by intraperitoneal injection of avertin (250 mg/kg) and placed in the prone position. Hair was removed from both hindlimbs with electric clippers, and the skin was disinfected with 70% ethanol. To completely eliminate any confounding variables associated with anatomical laterality, the injury was performed bilaterally. A gluteal incision was made, and both sciatic nerves were exposed using a transgluteal approach by blunt separation of the biceps femoris. The sciatic nerves on both sides were isolated from surrounding tissue and subjected to a standardized crush injury with fine forceps for 30 sec at the mid-thigh level, approximately 5 mm distal to the sciatic notch. Care was taken to ensure uniform compression while minimizing damage to adjacent tissues. The completeness of the crush was verified by visual inspection of nerve translucency. After the procedure, the muscle layers were repositioned, and the incision was closed with 6-O black silk sutures. Sham-operated mice underwent identical bilateral procedures including nerve exposure but without crush injury in the sciatic nerve. Postoperative analgesia was provided with ketoprofen (5 mg/kg, intramuscular injection). Mice were allowed to recover on a warming pad and monitored until fully ambulatory. Animal health and behavior were monitored daily. Humane endpoints were predefined, and animals were euthanized immediately or within the same day if they exhibited >20% body weight loss, reduced food intake for more than 2 days, respiratory distress, persistent abnormal behavior or physiological signs for more than 2 days, or a decrease in body temperature exceeding 4°C. No animals died or met humane endpoint criteria during the experiment.

### Tissue harvest, processing, and blinding

After 7 days, mice were anesthetized by intraperitoneal injection of avertin (250 mg/kg) and transcardially perfused with 1 × phosphate-buffered saline (PBS). The tibialis anterior, extensor digitorum longus, gastrocnemius, soleus, and plantaris muscles were harvested bilaterally. Left-side muscles were snap-frozen in isopentane precooled in liquid nitrogen, embedded in OCT compound [15,16], stored at −80°C, and sectioned at 5 µm using a cryostat. Right-side muscles were fixed in 10% neutral-buffered formalin overnight, paraffin-embedded, and sectioned at 5 µm. Both FFPE sections and cryosections were stained with H&E [17], TB [18], or WGA [19]. H&E- or TB-stained sections were digitized using a Magscanner KF-PRP-005 slide scanner (KFBIO technology for health, Yuyao, China) at 40 × magnification to obtain high-resolution whole-slide images. Images of WGA-stained sections were acquired using an Olympus BX51 microscope equipped with a DP71 digital camera (Olympus, Tokyo, Japan) using a 40 × objective lens. To prevent human bias, investigators were strictly blinded to the experimental groups and injury status during tissue processing and whole-slide image (WSI) acquisition.

### Hematoxylin and eosin (H&E) staining

For paraffin-embedded sections, automated staining was performed using the Leica HistoCore SPECTRA ST system (Leica Biosystems, Wetzlar, Germany). Sections were deparaffinized in xylene (four 1-min changes) and rehydrated through a descending ethanol series (100% to 95%). After rinsing in tap water for 2 min, nuclear staining was performed with Harris hematoxylin (twice, 2 min each), followed by differentiation in 0.5% HCl for 5 sec and rinsing for 1.5 min. Sections were blued in 1% ammonia water for 10 sec and rinsed for 2 min. Counterstaining was performed with eosin Y for a total of 25 sec (10 sec and 15 sec steps). Following dehydration in an ascending ethanol series and clearing in xylene, slides were mounted with a permanent mounting medium. For cryosections, automated staining was conducted using the Leica ST 5020 system (Leica Biosystems) with a modified protocol: sections were fixed in 95% ethanol (10 sec), rinsed, and stained with hematoxylin (three 8-sec steps). After rinsing, sections were counterstained with eosin Y for 8 sec, dehydrated, cleared, and mounted with a permanent medium.

### Toluidine blue (TB) staining

A 0.04% TB staining solution was prepared by diluting a 1% stock solution in 0.1 M sodium acetate buffer (pH 4.0) and filtering through a 0.45 µm membrane filter before use. TB staining was performed manually as follows. Paraffin sections were deparaffinized in xylene (three 4-min changes) and rehydrated through 95% and 70% ethanol. Sections were stained with the TB staining solution for 5 min, rinsed in tap water for 1 min, and dried at room temperature. Finally, slides were cleared in xylene for 5 min and mounted with a resin-based mounting medium. For cryosections, the staining procedure was identical, except that the deparaffinization and rehydration steps were omitted.

### Wheat germ agglutinin conjugated with Alexa Flour 555 (WGA) staining

Paraffin sections were deparaffinized in xylene three times for 4 min and rehydrated through 95% and 70% ethanol. To prevent non-specific binding, sections were blocked with 5% bovine serum albumin (BSA) in Tris-buffered saline (TBS) for 20 min. Following the blocking step, sections were incubated with 10 µg/ml WGA in 1% BSA in TBS at 37°C for 1 h. The slides were washed in TBS twice for 3 min. Finally, the sections were mounted using Dako fluorescence mounting medium.

### Image preparation and downsampling optimization

Digitized WSIs acquired via the scanner were initially reviewed in QuPath (version 0.6.0) [20]. Images were quality-controlled prior to export to ensure: i) adequate focus across the entire field, ii) uniform staining intensity, and iii) the absence of tissue folding or tears in the region of interest. Following this comprehensive quality control (QC) review, the soleus muscle was exclusively selected for all subsequent automated morphometric analyses in the present study. This muscle serves as an ideal model because its robust response to sciatic nerve crush injury is well-documented [21], and its relatively small anatomical size allows the entire cross-section to be captured within a single, contiguous image without artifactual fragmentation.

To determine the optimal spatial resolution for myofiber segmentation, whole cross-sectional images of the mid-belly region of the soleus muscle from seven mice (*n* = 7) were used. QuPath images at a native resolution of 4.00 px/µm were systematically downsampled to generate a multi-resolution dataset: D1 (4.00 px/µm), D2 (2.00 px/µm), D4 (1.00 px/µm), D8 (0.50 px/µm), and D16 (0.25 px/µm). The resolution of D4 was selected as the operational reference because it effectively balanced morphological detail with the suppression of intracellular textural noise (e.g., myofibrillar patterns) that can impair deep-learning performance in digital pathology [22]. Segmentation performance across resolutions was evaluated against the D4 reference using spatial overlap metrics (Dice coefficient and Intersection over Union (IoU)) [23,24] and morphometric stability.

Consequently, whole-section images were exported directly from QuPath utilizing this optimized downsampling factor of 4 (D4). Because the entire cross-section was processed as a single image, no image tiling or splitting was necessary, thereby eliminating the need for complex aggregation or double-counting corrections. Images were exported as standard formats (JPG, PNG, or TIFF), yielding final resolutions generally ranging from 0.8 to 1.0 px/µm depending on the specific native scan. To ensure absolute transparency, the exact spatial resolution and image size for each analyzed section are detailed in the respective figure legends.

### Automated myofiber segmentation using web-based Cellpose-SAM

Automated muscle fiber segmentation was performed for each single whole-section image using the Cellpose-SAM web interface (https://huggingface.co/spaces/carsen/cellpose-sam, version 1.2.0). Specifically, the interface utilizes the unified Cellpose-SAM model (identifier: cpsam), which is built upon the Segment Anything Model’s ViT-L (Vision Transformer-Large) backbone architecture. Exported images (regularly 5–20 MB, well within the 250 MB platform limitation) were uploaded, and the default segmentation parameters were applied without modifications: max resize = 1000, max iter = 250, flow thresh = 0.4, and cellprob thresh = 0. After automatic segmentation, the resulting masks files were downloaded. The average processing time was rapid, taking 7.5 ± 2.1 seconds per image when evaluated across 50 representative skeletal muscle images during the testing phase.

### Pixel (px) calibration, morphological filtering, and CSA measurement

Both the original images and their corresponding downloaded masks files were imported into Fiji/ImageJ (version 2.16.0/1.54p) [25]. ROI generation, morphological filtering, and measurement of myofiber CSA were then performed using a custom macro for automated batch processing. To ensure strict measurement integrity, the exact pixel (px)-to-micron (μm) calibration, intrinsically derived from the scanner’s optical metadata and the downsampling factor, was uniformly enforced using the Set Scale command. A manual QC step confirmed this calibration accuracy, demonstrating a highly reliable measurement tolerance of less than 1%. Following scale verification, the custom macro was executed, prompting the input of the validated scale, size limits (100–4000/6000/10000 μm^2^, depending on the image), and a circularity threshold (≥ 0.3). Based on these parameters, filtering was applied to eliminate non-myofiber regions and artifacts. Furthermore, as a crucial part of the morphological filtering, the “Remove Border Labels” function from the MorphoLibJ plugin (version 1.2.0) [26] was utilized. This automatically and completely excluded any incomplete boundary myofibers touching the extreme edges of the single whole-section image, supporting accurate CSA quantification while reducing artifactual bias. Finally, while the initial myofiber segmentation was efficiently automated by the Cellpose-SAM model, all resulting masks were rigorously inspected. Any improperly segmented boundaries (e.g., falsely merged or split fibers) were manually corrected by investigators strictly blinded to the experimental groups prior to the final extraction of CSA data, thereby reducing the risk of subjective bias.

### Code availability, long-term reproducibility, and data availability

The custom ImageJ macro file is freely available on GitHub (https://github.com/kimjr0/myofiber-csa), with detailed usage instructions provided in the Supporting Information. To ensure long-term computational reproducibility and mitigate the risks associated with relying solely on third-party hosted web applications, we developed a local, scriptable Python alternative. In accordance with open science practices, all underlying materials have been deposited and permanently archived. This includes our dataset (raw data in Excel format and representative input images) alongside the computational pipeline (the Python script, custom ImageJ filtering macro, and comprehensive user documentation). All files are publicly accessible via Zenodo (https://doi.org/10.5281/zenodo.21482173). Detailed experimental protocols are publicly available through protocols.io (https://dx.doi.org/10.17504/protocols.io.36wgq2jx3gk5/v1).

### Quantitative validation of segmentation accuracy

The reliability of the web-based Cellpose-SAM (CS) workflow [9,11] was validated against manual ground-truth annotations performed by two investigators using 10 representative images (*n* = 10) selected from the soleus muscle sections of seven mice (1–2 representative images per section) stained with H&E or WGA. Prior to segmentation, the original images were downsampled by a factor of 4 in QuPath and converted to 8-bit grayscale to optimize boundary detection. Segmentation accuracy was assessed using the Dice coefficient and IoU. The manual-to-manual (M-M) comparison was based on *n* = 10 image pairs (one pair for each of the 10 representative images), whereas the CS-to-manual (CS-M) comparison was based on *n* = 20 image pairs, with the CS segmentation from each image compared against the corresponding annotations generated by both investigators. To evaluate morphometric consistency, individual myofiber ROIs were extracted using Fiji/ImageJ for precise CSA calculation. Additionally, the broad robustness of the pipeline was confirmed by comparing the mean CSA measurements and CSA distributions derived from H&E- and WGA-stained images (*n* = 10).

### Statistical analysis

Data are expressed as mean ± SEM. Statistical differences between two groups were analyzed using a two-tailed unpaired Student’s *t*-test, whereas comparisons among multiple groups were evaluated using a one-way analysis of variance (ANOVA) followed by Tukey’s post hoc test. The Kolmogorov-Smirnov (KS) test was used for descriptive comparison of pooled fiber-level CSA distributions and was not used for biological inference. A *p* value of < 0.05 was considered statistically significant. Data visualization and statistical analyses were conducted using GraphPad Prism 8 (GraphPad Software, San Diego, CA, USA).

## Results

### Feasibility of the web-based Cellpose-SAM (CS) workflow on H&E-stained FFPE tissue sections

**Fig 1:**
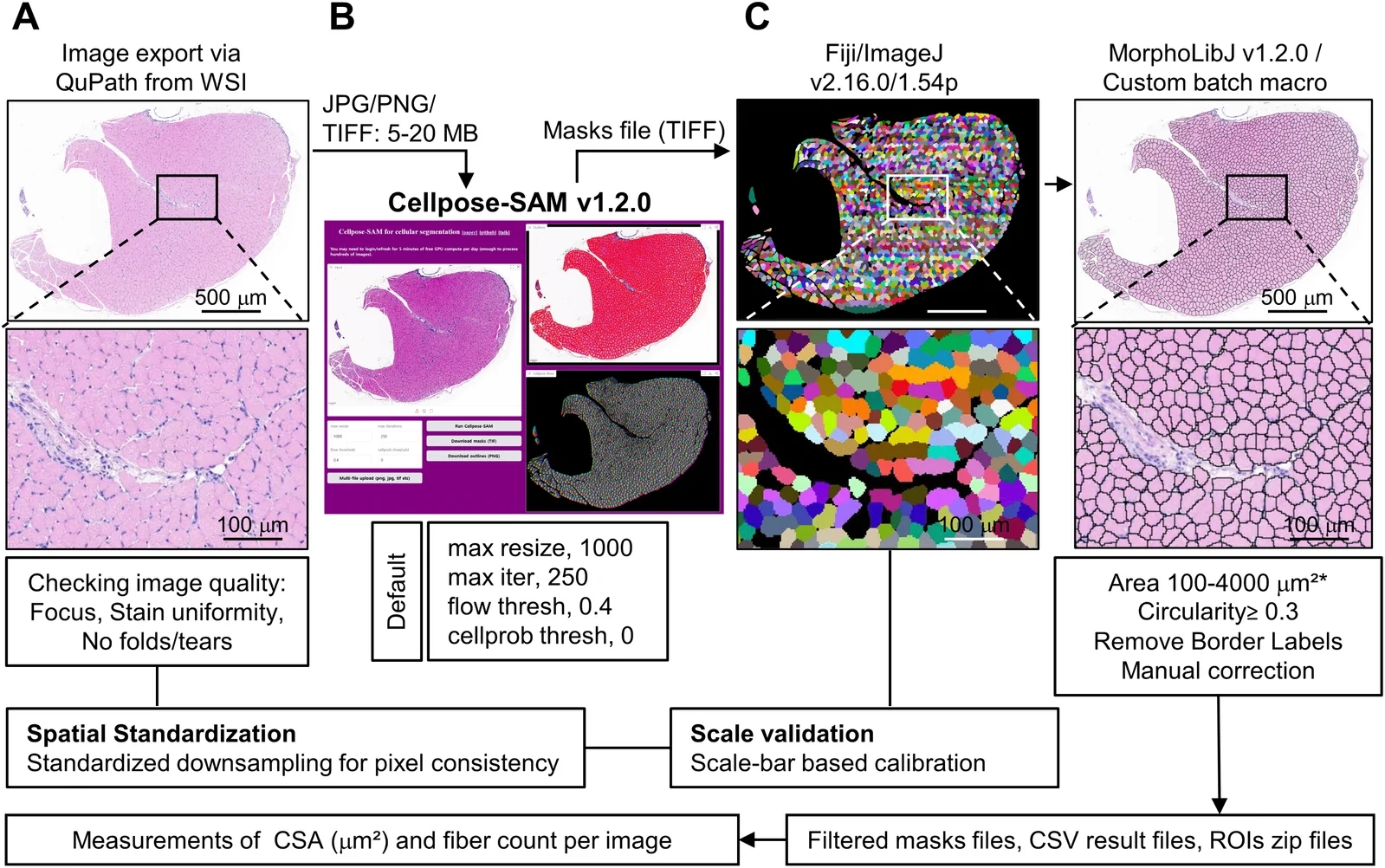
Experimental scheme of muscle fiber cross-sectional area (CSA) analysis using H&E staining and the web-based Cellpose-SAM (CS) workflow. The analytical pipeline consists of three main sequential stages. **A**. Image export via QuPath from a whole-slide image (WSI). High-resolution images are selected from WSI and exported with standardized downsampling for pixel consistency, following strict quality control (QC) criteria (focus, stain uniformity, and absence of tissue folds/tears, etc). The boxed region indicates the area shown at higher magnification below. **B**. Cellpose‑SAM web interface used for automated myofiber segmentation. Image files (5-20 MB) are processed via the web-based CS interface (version 1.2.0) in https://huggingface.co/spaces/mouseland/cellpose with default parameters (max resize, 1000; max iter, 250; flow thresh, 0.4; cellprob thresh, 0). **C**. Postprocessing and measurements of CSA and total fiber count. Original image and downloaded masks image are imported into Fiji/ImageJ (v1.54p). Following scale (px/µm) verification, a custom batch macro was executed. Within the macro, the scale, size limits (100-4000 µm^2^), and circularity threshold (≥ 0.3) were inputted. *The upper limit for the CSA exclusion criterion was specifically adjusted (ranging from 4,000 to 10,000 µm^2^) depending on the biological characteristics of the experimental groups. The boxed region indicates the area shown at higher magnification below. The macro utilized MorphoLibJ to filter out non-myofiber regions and border-touching myofibers. The resulting ROIs were overlaid onto the images for manual inspection and correction. Once the final ROIs were confirmed, the macro quantified the CSA and total fiber count, saving the final outputs as CSV result files, alongside the filtered ROIs and masks files. Enlarged region showing clean, artifact‑filtered ROIs outlining individual myofibers suitable for quantitative morphometry.

To assess the feasibility of our workflow on H&E-stained FFPE muscle sections, we first established a standardized pipeline integrating image export from WSIs, automated segmentation, and morphometric postprocessing (Fig 1). The workflow began by importing the WSIs into QuPath. Following image quality control, regions of interest (ROIs) were exported with standardized downsampling to ensure spatial consistency and uniform pixel resolution across all samples. High-magnification views of the exported sections confirmed well-preserved fiber architecture and clearly defined myofiber boundaries (Fig 1A). For myofiber segmentation, we utilized the web-based CS interface, which eliminates the need for high-end GPU hardware or complex local software installations (Fig 1B). The exported H&E images were processed using default segmentation parameters without any modifications, and the resulting label masks files were downloaded. Postprocessing was performed in Fiji/ImageJ (v1.54p) utilizing the MorphoLibJ (v1.2.0) plugin and a custom macro (Fig 1C). Both the original images and their corresponding downloaded masks files were imported into the software. Following scale (px/µm) verification, the custom macro was executed, prompting the input of the validated scale, size limits (100–4000 µm^2^), and circularity threshold (≥ 0.3). Based on these parameters, MorphoLibJ filtering was applied to eliminate non-myofiber regions, artifacts, and border-touching myofibers. The generated ROIs were then overlaid onto the original images, allowing for manual inspection and correction. This process yielded clean, measurement-ready ROIs sets. Once the final ROIs were confirmed, the macro automatically quantified the CSA and total fiber count per image. Finally, the quantified data were exported as a comma-separated values (CSV) file, alongside the filtered ROI zip files and masks files. Collectively, these findings demonstrate that the web-based CS workflow is fully compatible with standard H&E-stained FFPE muscle sections, suggesting its feasibility and efficiency for high-throughput, routine morphometric analysis.

### Optimization of image resolution to establish a robust operational range for web-based CS myofiber segmentation in H&E-stained tissues

**Fig 2:**
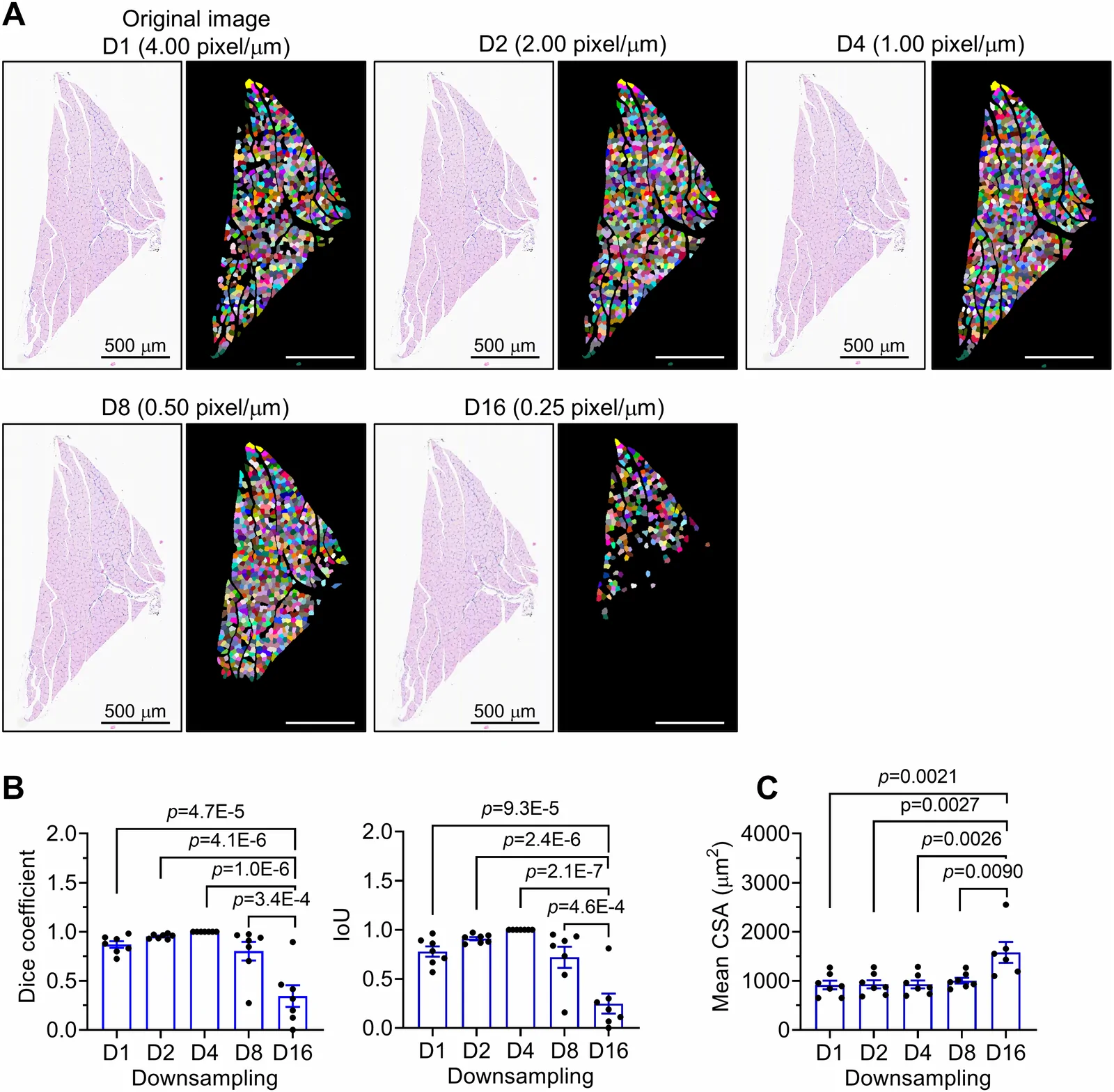
Optimization of image resolution for the web-based Cellpose-SAM (CS) myofiber segmentation. All analyses were performed using whole cross-sectional images obtained from the mid-belly region of the soleus muscle from seven mice (*n* = 7). **A**. Representative H&E-stained downsampled images (D1 to D16) and their corresponding segmentation masks. Images were progressively downsampled from the native resolution (D1) to D16. The specific resolutions and dimensions are as follows: D1 (4.00 px/µm, 6134 × 10003 px), D2 (2.00 px/µm, 3067 × 5004 px), D4 (1.00 px/µm, 1534 × 2502 px), D8 (0.50 px/µm, 767 × 1251 px), and D16 (0.25 px/µm, 383 × 626 px). **B**. Quantitative evaluation of segmentation performance across different downsampling scales using the Dice coefficient (left) and Intersection over Union (IoU) (right). The optimal segmentation accuracy was achieved at D4 (1.00 px/µm), which demonstrated significantly higher spatial overlap compared to other resolutions. **C**. Morphometric comparison of mean CSA across the downsampled groups. Data are presented as mean ± SEM. Statistical significance was determined by one-way ANOVA, followed by Tukey’s multiple comparisons test.

To identify the optimal resolution for H&E-stained sections, we evaluated the performance of the web-based CS pipeline across a multi-resolution dataset comprising whole cross-sectional images obtained from the mid-belly region of the soleus muscle in seven mice (*n* = 7 for all analyses). Representative original H&E images ranging from the native resolution (D1) to the lowest resolution (D16), along with their corresponding automated segmentation masks, are presented in Fig 2A. Based on these visual representations, we performed a quantitative analysis of spatial overlap, which revealed that D4 (1.00 px/µm) is the most accurate scale (Fig 2B). Peak performance occurred at D4, exhibiting the highest Dice coefficient and IoU, which were significantly superior to all other downsampling groups. The native resolution (D1; 4.00 px/µm) and D2 (2.00 px/µm) showed slightly lower concordance, likely due to potential over-segmentation triggered by intracellular textural noise. Conversely, lower resolutions suffered from under-segmentation and fiber merging as interstitial boundaries became progressively blurred; notably, both the Dice score and IoU for D16 (0.25 px/µm) dropped sharply. Furthermore, morphometric analysis of the mean CSA directly supported this segmentation trend (Fig 2C). The mean CSA remained highly stable with no significant differences observed among the D1, D2, D4, and D8 groups. However, the D16 group exhibited a significant, artifactual increase in the mean CSA compared to all other groups, which closely reflects the severe fiber merging and mask dropouts observed previously. Taken together, these findings establish an optimal resolution at approximately 1 px/µm (D4), while maintaining a robust operational plateau between 0.5 and 4 px/µm (D8 to D1), providing a highly tolerant analytical standard for varying imaging platforms.

### Quantitative validation of segmentation accuracy and cross-stain reliability

**Fig 3:**
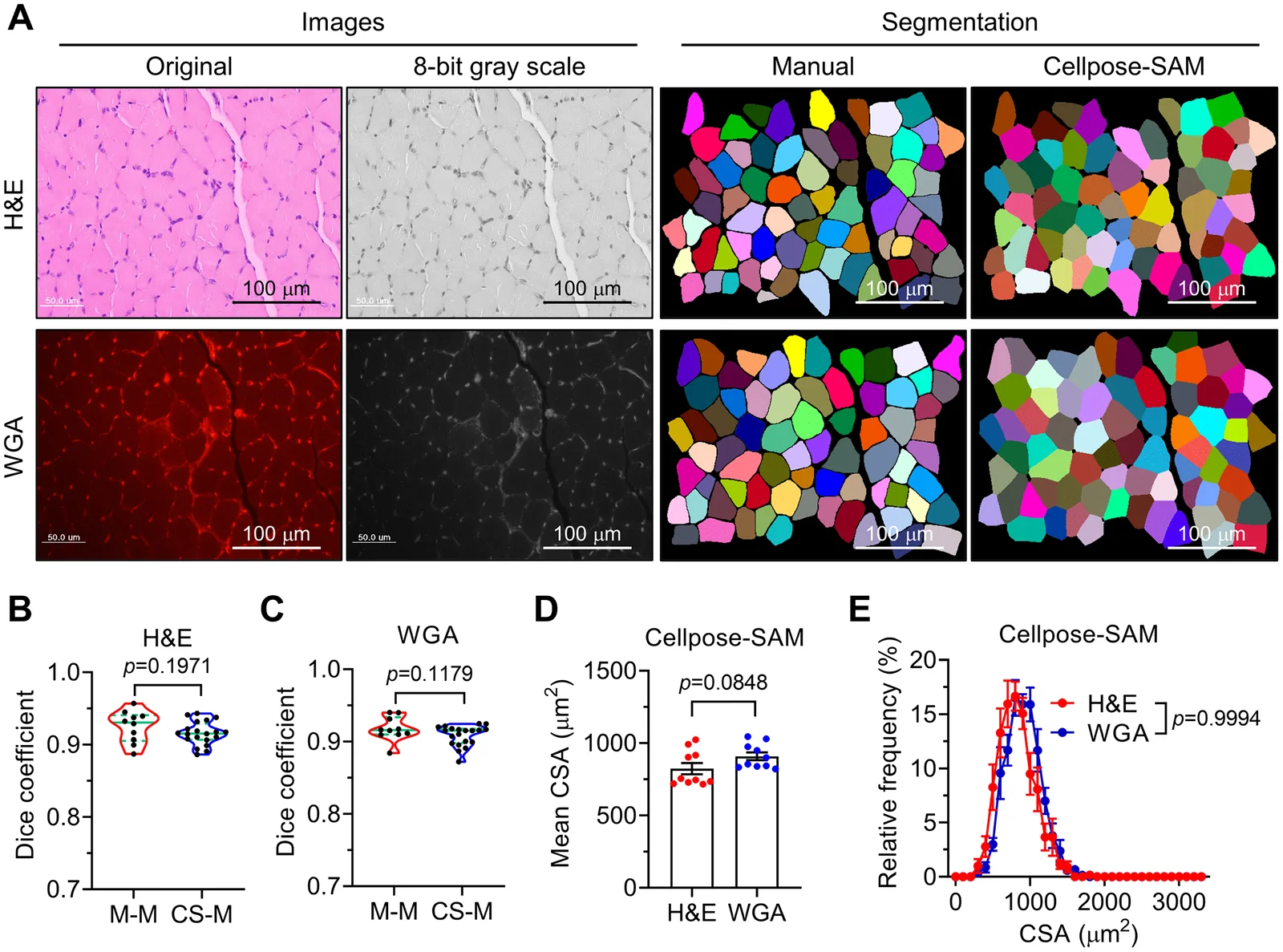
Quantitative comparison of the web-based Cellpose-SAM (CS) and manual (M) segmentation in H&E and WGA-stained sections. Analyses were performed using 10 representative images (*n* = 10) selected from the soleus muscle sections of seven mice (1-2 representative images per section). **A**. Representative images of H&E and WGA-stained sections (image resolution, 1.4 px/µm; size, 510 × 384 px), displaying manual ground truth and CS-generated masks. **B**. Dice coefficients for manual-to-manual (M-M, *n* = 10 image pairs) and CS-to-manual (CS-M, *n* = 20 image pairs from the same 10 images) comparisons for H&E-stained sections. **C**. Dice coefficients for manual-to-manual (M-M, *n* = 10 image pairs) and CS-to-manual (CS-M, *n* = 20 image pairs from the same 10 images) comparisons for WGA-stained sections. Data are presented as violin plots showing individual data points, with solid green lines representing the median and dashed green lines indicating the quartiles. **D**. Comparison of the mean CSA between H&E (*n* = 10) and WGA (*n* = 10) images, which were derived from consecutive tissue sections and analyzed by the web-based CS pipeline. Data are expressed as mean ± SEM. **E**. Relative frequency distributions of the CSA derived from the same images analyzed in D. The distributions represent pooled fiber-level CSA values and are presented for descriptive comparison of myofiber CSA patterns between staining modalities. Statistical significance was evaluated by two-tailed unpaired Student's *t*-tests (B, C, and D) and the Kolmogorov-Smirnov test (E).

To validate the reliability of the web-based CS pipeline on routine H&E-stained images, we compared its segmentation performance against manual (M) tracing using the Dice coefficient and IoU. Prior to segmentation, the original images were converted to 8-bit grayscale to standardize the input. Representative images illustrating this workflow, comprising the original images, 8-bit grayscale conversions, and the highly concordant segmentation masks generated by both manual tracing and the web-based CS pipeline, are shown in Fig 3A. To establish a baseline for comparison, manual annotations of H&E-stained images (*n* = 10 image pairs) were performed by two investigators (M1 and M2). The manual-to-manual (M-M) baseline was defined as the agreement between M1 and M2 (*n* = 10 images). Subsequently, the automated CS outputs for the same 10 images were compared against both M1 and M2, yielding a total of 20 image pairs for the CS-to-manual (CS-M) group. There was no significant difference in the Dice coefficient (*p* = 0.1971; Fig 3B) and the IoU (*p* = 0.8170; S1A Fig) between the M-M baseline and the CS-M segmentation, suggesting that the web-based CS segmentation performance falls within the range of inter-observer variability. The distributions of the Dice coefficient and IoU for the M-M and CS-M comparisons are summarized in Table 1 as the median and interquartile range (Q1-Q3).

**Table 1:** Comparison of Dice coefficient and IoU values between web-based Cellpose-SAM (CS) and manual (M) segmentation in H&E- and WGA-stained sections. Values are presented as median and interquartile range (Q1-Q3).

Stain	Comparison	Dice coefficient, Median (Q1-Q3)	IoU, Median (Q1-Q3)
H&E	M-M^a^	0.9306 (0.9094-0.9388)	0.8512 (0.8375-0.8756)
	CS-M^b^	0.9154 (0.9075-0.9243)	0.8452 (0.8296-0.8739)
WGA	M-M	0.9159 (0.9103-0.9278)	0.8448 (0.8354-0.8653)
	CS-M	0.9151 (0.8988-0.9171)	0.8434 (0.8163-0.8469)

^a^M-M: Comparison between manual segmentations performed by two annotators (*n* = 10 image pairs).^b^ CS-M: Comparison between Cellpose-SAM (CS) segmentation and each manual annotation (*n* = 20 image pairs derived from the same 10 images).

While routine H&E staining provides sufficient morphological context, precise delineation of the sarcolemma or extracellular matrix (ECM) is traditionally validated using immunohistochemical staining for proteins such as laminin or collagen IV. However, these markers are largely restricted to cryosections due to epitope masking and degradation during FFPE procedures. Therefore, to establish a robust reference standard in FFPE tissues, we utilized WGA, a lectin that reliably binds to sarcolemmal glycoproteins and ECM, allowing for distinct visualization of true myofiber boundaries [19] (Fig 3A).

Applying the identical experimental design to these WGA-stained images (yielding 10 M-M and 20 CS-M image pairs), the automated segmentation similarly performed comparably to manual tracing, exhibiting no significant difference in the Dice coefficient (*p* = 0.1179; Fig 3C) and the IoU (*p* = 0.1121; S1B Fig).

Furthermore, we investigated whether the choice of staining method affects the ultimate morphometric outcome. The CS pipeline revealed no significant differences in image-level mean CSA between H&E- and WGA-stained images (*p* = 0.0848, *n* = 10 images; Fig 3D). The pooled fiber-level CSA distributions also showed similar distribution patterns (KS test, *p* = 0.9994, *n* = 10 images); this comparison is presented for descriptive purposes only (Fig 3E). Taken together, these results demonstrate that the web-based CS pipeline provides reliable myofiber segmentation while reducing subjective bias across different staining modalities in FFPE tissues.

### Concordance between H&E and TB for CSA distributions

**Fig 4:**
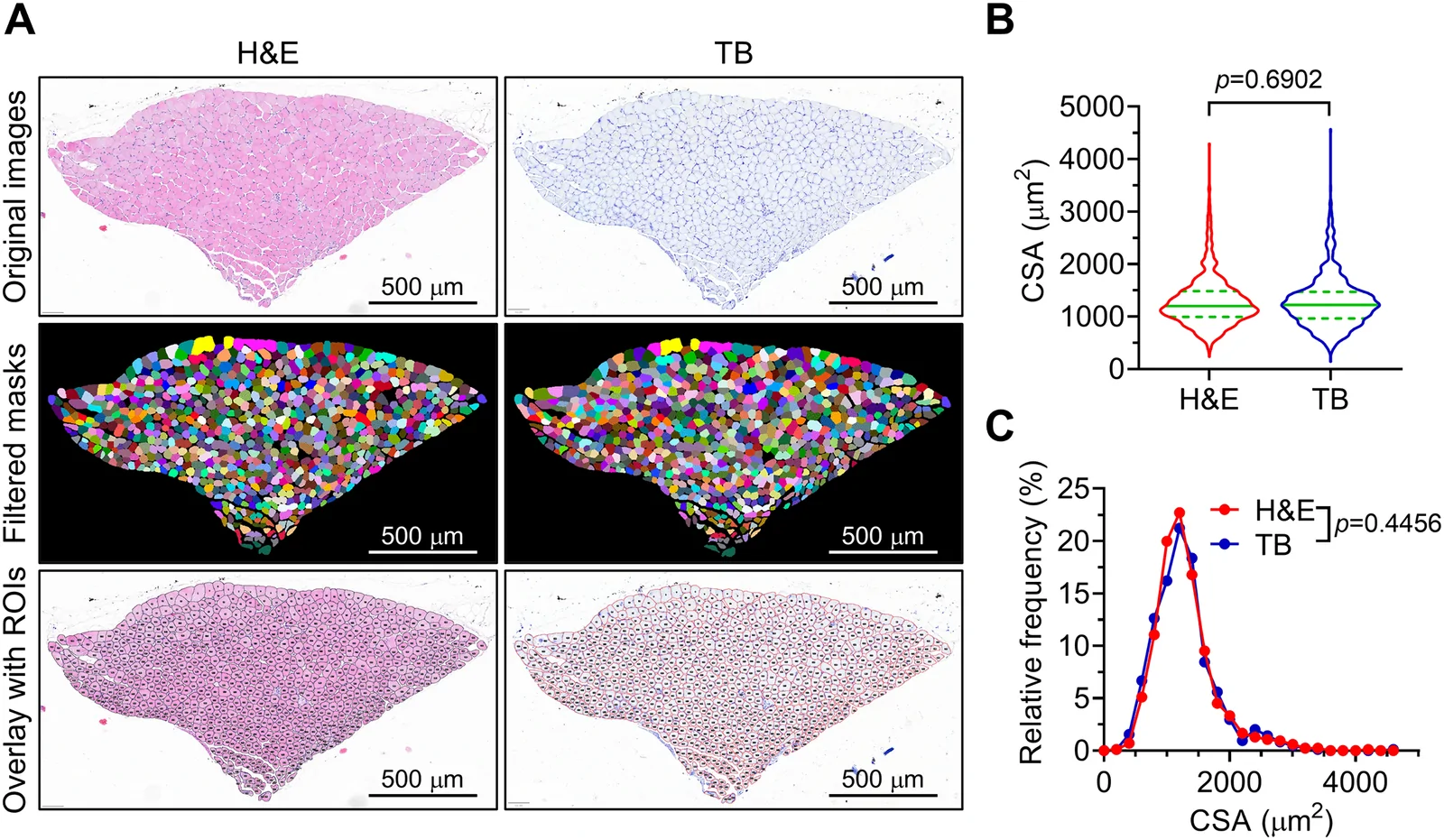
Comparison of myofiber cross-sectional area (CSA) from FFPE sections stained with H&E or TB using Cellpose‑SAM segmentation. **A**. Representative FFPE soleus muscle sections stained with H&E (image resolution, 1.0 px/μm; size 2219 × 1166 px) or TB (image resolution, 1.0 px/μm; size 2184 × 1135 px). Corresponding Cellpose‑SAM segmentation outputs are shown as filtered masks images (area, 100-6000 μm^2^), along with overlay images displaying the final ROI boundaries on the original histological sections. **B**. Quantification of myofiber CSA obtained from H&E‑ and TB‑stained FFPE soleus sections. Data are presented as violin plots with solid green lines representing the median and dashed green lines indicating the quartiles. The violin plots represent pooled fiber-level CSA distributions and are presented for descriptive comparison between staining modalities. Statistical significance was evaluated by two-tailed unpaired Student's *t*-tests. **C**. Frequency distribution of CSA values derived from H&E and TB images, presented for descriptive comparison.

To verify that our workflow is appropriate for H&E‑stained FFPE tissue, we first applied the analysis to whole soleus muscle sections. Cellpose‑SAM produced accurate myofiber segmentation from H&E images, and CSA values were reliably quantified across the entire section (Fig 4A). We then assessed whether staining method influenced the results by comparing H&E‑ and TB‑stained FFPE sections (Fig 4A). The pooled fiber-level CSA distributions obtained from H&E- and TB-stained images showed comparable patterns, with mean CSA values of 1288.66 ± 479.05 μm^2^ (*n* = 841 fibers) and 1279.20 ± 493.50 μm^2^ (*n* = 839 fibers), respectively (Fig 4B). Consistent with these findings, CSA distributions and total fiber numbers showed no significant differences between H&E- and TB-stained sections (Fig 4C).

**Fig 5:**
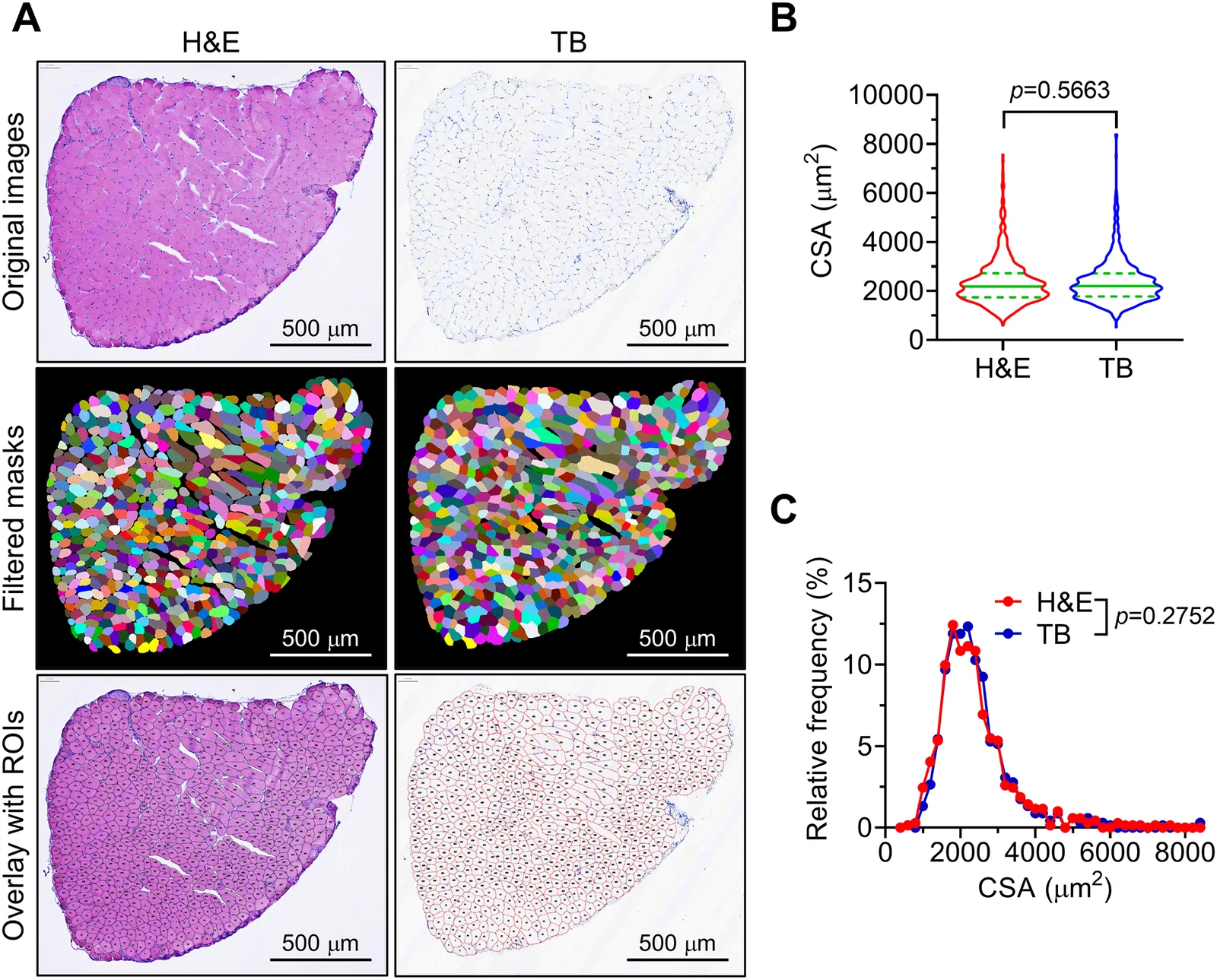
Comparison of myofiber cross‑sectional area (CSA) from cryosections stained with H&E or TB using Cellpose‑SAM segmentation. **A**. Representative cryosections of whole soleus muscle stained with H&E (image resolution, 1.0 px/μm; size, 1785 × 1534 px) or TB (image resolution, 1.0 px/μm; size, 1888 × 1557 px). Corresponding Cellpose‑SAM segmentation outputs are shown as pseudocolor label maps (area, 100-10000 μm^2^), followed by overlay images displaying final ROI boundaries superimposed on the original histological sections. All images demonstrate robust segmentation performance across staining types. **B**. Quantification of myofiber cross‑sectional area (CSA) obtained from H&E‑ and TB‑stained cryosections. Data are presented as violin plots with solid green lines representing the median and dashed green lines indicating the quartiles. The violin plots represent pooled fiber-level CSA distributions and are presented for descriptive comparison between staining modalities. Statistical significance was evaluated by two-tailed unpaired Student's *t*-tests. **C**. Frequency distributions of CSA values derived from H&E and TB cryosections, presented for descriptive comparison.

We next evaluated whether cryosections exhibited the same staining concordance observed in FFPE tissue. In cryosectioned soleus muscle, Cellpose‑SAM produced highly consistent segmentation across both staining methods, with no detectable differences in median CSA, fiber count, or overall CSA distributions between H&E‑ and TB‑stained sections (Fig 5A). The pooled fiber-level CSA measurements showed comparable mean values between staining methods, measuring 2352.50 ± 946.09 μm^2^ (*n* = 692 fibers) for H&E and 2381.26 ± 911.42 μm^2^ (*n* = 681 fibers) for TB (Fig 5B). Violin plots demonstrated overlapping central tendencies and dispersion, and relative frequency curves showed closely matched profiles (Fig 5B-5C), consistent with comparable segmentation performance in cryosections. Notably, cryosections displayed substantially larger mean CSA values than FFPE sections (approximately 2352.50 μm^2^ vs. 1288.66 μm^2^), consistent with the expected tissue shrinkage associated with formalin fixation and paraffin embedding [27].

Together, these results indicate that our workflow is well suited for myofiber CSA measurements in both FFPE sections and cryosections stained with H&E, and that H&E and TB staining provide comparable segmentation performance and CSA quantification under the conditions tested.

### Detecting denervation‑induced atrophy using the web-based CS workflow

**Fig 6:**
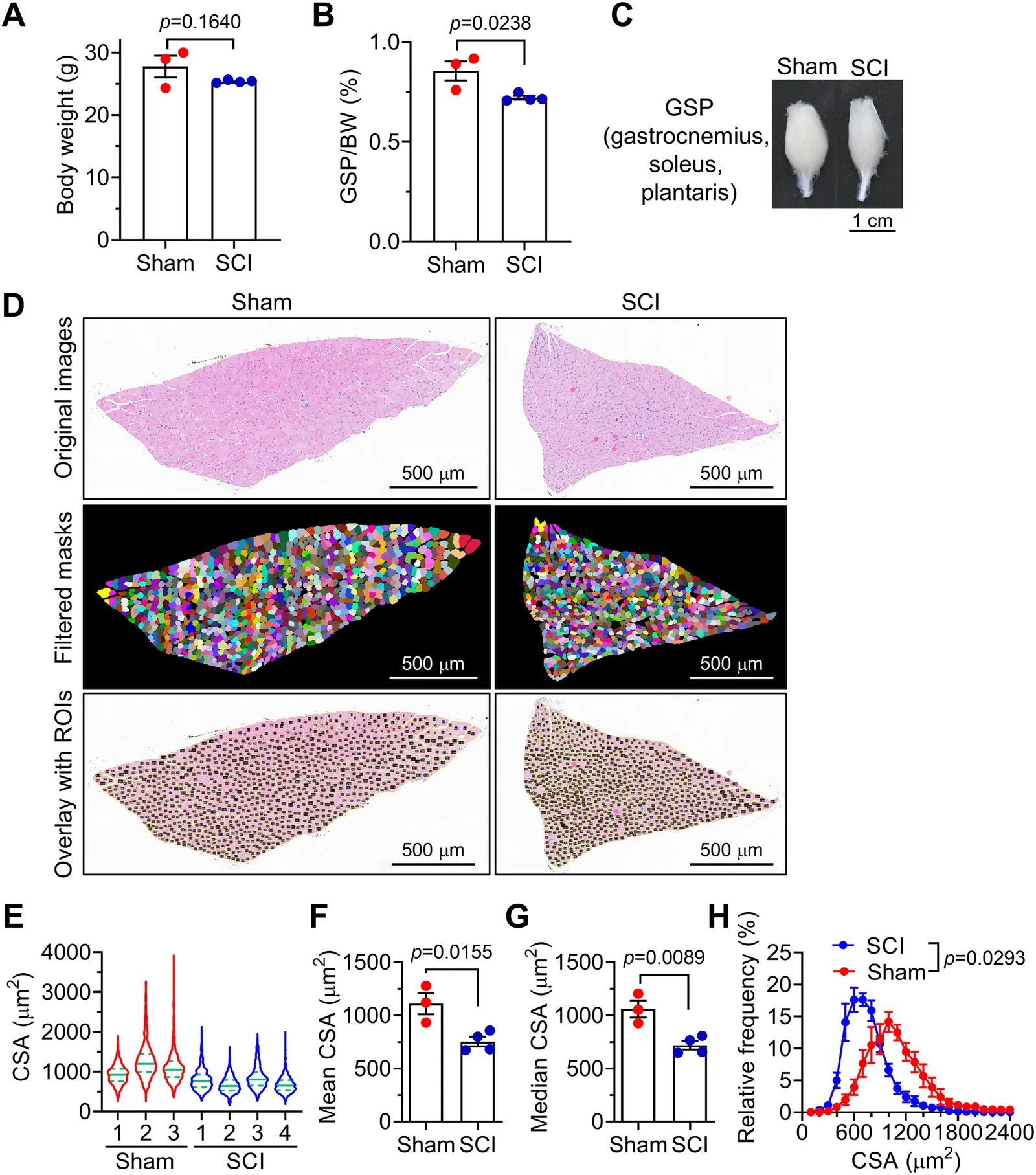
Analysis of myofiber cross‑sectional area (CSA) in soleus muscle after sciatic nerve crush injury (SCI). **A**. Body weights of sham and SCI mice (sham, *n* = 3 mice; SCI, *n* = 4 mice). **B**. Ratios of gastrocnemius, soleus, and plantaris (GSP) muscle weights to body weight in sham and SCI groups. **C**. Representative gross images of GSP muscles from sham and SCI groups. **D**. Representative H&E‑stained FFPE sections of soleus muscles from sham and SCI mice. The images were acquired at a constant resolution of 0.8 px/μm, with image dimensions of 2442 × 1457 px for the sham and 1830 × 996 px for the SCI. Corresponding Cellpose‑SAM segmentation outputs, including pseudocolor masks images and ROI overlays generated after filtering and manual correction. For panels **E-H**, whole cross-sectional images of the mid-belly region of the soleus muscle were analyzed, with one image from each animal (sham, *n* = 3 mice; SCI, *n* = 4 mice). **E**. Quantification of soleus myofiber CSA for individual mice. Data are presented as violin plots with solid green lines representing the median and dashed green lines indicating the quartiles. **F**. Group comparison of per-animal mean CSA between sham and SCI groups. **G**. Group comparison of per-animal median CSA between sham and SCI groups. **H**. Frequency distribution of CSA values from sham and SCI groups, presented for descriptive comparison. Data are expressed as mean ± SEM. Statistical significance was determined by a two-tailed unpaired Student’s *t*-tests (A, B, F, and G) and the Kolmogorov-Smirnov test (H).

To evaluate the workflow’s ability to detect biologically meaningful changes in muscle morphology, we analyzed soleus muscle from mice subjected to sciatic nerve crush injury (SCI, *n* = 4 mice) and sham surgery (*n* = 3 mice). At 7 days post‑injury, body weight did not differ between groups (Fig 6A), whereas the gastrocnemius-soleus-plantaris (GSP) muscle weight normalized to body weight was significantly reduced in SCI mice (*p* = 0.0238; Fig 6B), indicating early denervation‑induced atrophy. Representative gross images further demonstrated visible muscle mass loss in the SCI group (Fig 6C). H&E‑stained FFPE soleus sections from sham and SCI mice were subsequently processed using the web-based CS workflow, which generated robust whole‑section myofiber segmentation masks and artifact‑filtered ROI sets appropriate for CSA quantification (Fig 6D). To accurately assess morphological changes in the soleus muscle, we analyzed the total fiber count and CSA for each individual animal in the sham and SCI groups. Summary statistics for the CSA (mean ± SD, median, Q1, and Q3) are provided in Table 2. Detailed values regarding the fiber analysis process, including the number of initially filtered fibers, the final number of analyzed fibers, manual corrections, exclusions, and the overall manual correction rate (%), are summarized in Supplementary Table 1. The soleus CSA of each individual animal was evaluated using violin plots (Fig 6E), which revealed a reduction in overall myofiber size in the SCI group compared to sham controls. In addition, comparison of the mean CSA values per animal confirmed a statistically significant decrease in the SCI group compared to the sham group (mean, 752.64 vs. 1109.88 μm^2^; 95% CI, −612.2 to −102.3; *p* = 0.0155; Fig 6F). Similarly, analysis of the median values also demonstrated a significant reduction (median, 718.75 vs. 1060.42 μm^2^; 95% CI, −553.5 to −129.8; *p* = 0.0089; Fig 6G). Analysis of the pooled fiber-level CSA distributions showed a leftward shift in the SCI group relative to the sham controls (KS test, *p* = 0.0293; Fig 6H); this comparison is presented for descriptive purposes only. This distributional pattern is consistent with the reductions in animal-level mean and median CSA (Figs 6F and 6G), reflecting the well‑established response to peripheral nerve injury, in which loss of neuromuscular innervation induces rapid myofiber atrophy within the first week of denervation [28]. Together, these findings demonstrate that the web-based CS workflow reliably detects biologically relevant changes in myofiber size distribution, enabling accurate identification of denervation‑induced atrophy from standard H&E‑stained FFPE muscle sections.

**Table 2:** Animal-level quantification of myofiber cross-sectional area (CSA) in the soleus muscle between sham and SCI groups.

Mouse number		CSA (μm²)	
		Mean ± SD	Q1, Median, Q3
Sham	1	931.99 ± 248.68	762.50, 925.00, 1075.00
	2	1276.47 ± 450.33	992.97, 1203.13, 1457.42
	3	1121.18 ± 407.51	868.75, 1053.13, 1264.06
SCI	1	800.27 ± 263.24	612.50, 764.84, 930.08
	2	672.96 ± 202.61	531.25, 646.88, 795.31
	3	857.19 ± 285.19	655.08, 808.59, 989.84
	4	680.13 ± 203.99	539.84, 654.69, 792.19

## Discussion

This study presents an installation‑free and broadly accessible workflow for quantifying myofiber CSA directly from routine bright‑field histology. By integrating web‑based CS segmentation with standardized morphological curation in Fiji/ImageJ, we demonstrate that reproducible morphometry can be performed on both FFPE sections and cryosections stained with H&E or TB. This capability reduces the dependency on immunofluorescence‑based laminin or collagen IV labeling and enables researchers to leverage the extensive FFPE archives commonly available in research and clinical laboratories.

In automated histological analysis, processing images at native high magnification often introduces heavy computational burdens without guaranteeing better accuracy. Our downsampling experiments demonstrated that the web-based CS pipeline performs best at a reduced resolution of approximately 1.0 px/μm. At higher resolutions, the model tended to over-segment fibers by misinterpreting intracellular textures as true borders. On the other hand, dropping the resolution too low (below 0.5 px/μm) blurred the extracellular matrix, causing adjacent fibers to merge and artificially inflating the mean CSA. Therefore, we recommend an optimal operational resolution of 0.8–2 px/μm for this workflow. This optimal resolution not only supports segmentation accuracy but also reduces computational burden, facilitating rapid and high-throughput morphometric screening.

Our findings indicate that the web-based CS workflow can perform automated myofiber segmentation with an accuracy comparable to expert annotation. Quantitative validation using the Dice coefficient and IoU revealed no significant differences between the CS outputs and manual ground-truth data. This high degree of spatial agreement suggests that the automated approach can streamline time-consuming manual processes while preserving the integrity of morphometric analysis across different histological preparations. Our analysis showed that routine H&E staining yielded CSA measurements comparable to those obtained using WGA fluorescent lectin staining, which served as a reliable reference method for FFPE tissues. These findings suggest that standard, cost-effective H&E staining can serve as a practical substitute for complex fluorescence-based labeling in automated morphometry.

Across datasets, segmentation performance and CSA measurements were comparable between H&E‑ and TB‑stained sections, confirming that commonly used bright‑field stains are comparable under the conditions tested for automated morphometry. Although FFPE samples exhibited the expected shrinkage relative to cryosections, this effect was systematic and did not appear to compromise within‑study comparisons [29]. Importantly, segmentation of comparable quality was achieved from H&E‑stained FFPE muscle, the most accessible specimen type, indicating that specialized staining is not required for reliable myofiber delineation in the present setting.

The workflow also demonstrated sensitivity to biologically meaningful changes in muscle morphology. In the sciatic nerve crush model, where denervation triggers rapid and heterogeneous fiber atrophy, the pipeline detected a significant reduction in mean CSA and a characteristic leftward shift in the fiber size distribution. The increased proportion of fibers below 800 μm^2^ observed following injury is consistent with known early responses to neuromuscular denervation [28,30]. That these changes were captured using only FFPE H&E sections underscores the practical utility of this approach for both experimental and clinical muscle pathology. Compared with fluorescence‑based approaches, this workflow offers several practical advantages. It eliminates the need for antibody optimization and specialized microscopy, substantially reduces processing time, and enables complete analysis within minutes. The use of open‑source Fiji macros supports reproducibility and scalability across datasets and research sites, lowering technical barriers for quantitative morphometry even in settings with limited computational resources.

Segmentation robustness was maintained across stain types and tissue preparations when three safeguards were applied: i) consistent color and contrast during image acquisition (or color normalization in Fiji/ImageJ), ii) exclusion of debris, vessels, and torn regions through attribute‑based filters, and iii) removal of partially imaged boundary fibers. Occasional over‑ or under‑segmentation in degenerating or regenerating zones was mitigated through watershed-based refinement in MorphoLibJ and manual correction by blinded investigators.

Several limitations should be considered when interpreting these findings. Cross-stain and cross-modality comparisons (H&E vs. TB and H&E vs. WGA) were performed using image- or animal-level summary measurements rather than direct one-to-one correspondence of individual myofibers. Although fiber-level matching across separately stained serial sections is technically feasible, achieving accurate one-to-one correspondence remains challenging because the same tissue section cannot generally be evaluated simultaneously with different staining protocols, requiring comparisons across adjacent serial sections that do not contain identical fiber profiles. Variations in sectioning plane, tissue deformation, and fiber orientation may further introduce uncertainty in fiber-level matching. Consequently, fiber-level agreement analyses, such as Bland-Altman analysis, which is commonly used to assess agreement between measurement methods [31], were not performed and remain a limitation of the current validation. As a partial substitute for direct fiber-level correspondence analyses, image-level mean CSA comparisons (Figs 3D, 4B, and 5B) and animal-level mean and median CSA measurements (Figs 6F and 6G) were used to provide complementary summary-level assessments of morphometric consistency and biological differences, respectively. In addition, the denervation injury model was evaluated using a limited number of animals (sham, *n* = 3; SCI, *n* = 4), and further validation in larger cohorts may be warranted. The current validation was performed using a single slide scanner, staining protocol, and mouse strain, and the performance of the segmentation workflow in severely dystrophic, necrotic, or actively regenerating muscle was not specifically evaluated. Furthermore, the primary workflow relies on a publicly hosted web interface, although a local Python implementation is provided as an alternative. Finally, this workflow was evaluated only in mouse soleus muscle, and its applicability to other muscles, species, and human archival tissue requires further investigation.

Overall, this lightweight and openly available workflow provides a practical and scalable approach for quantitative muscle morphometry, while the limitations described above should be considered when applying it to new experimental or clinical settings. Its generalizability to additional muscles and pathological contexts, such as muscular dystrophies, sarcopenia, cachexia, and metabolic myopathies, warrants further investigation. Future studies may incorporate fiber‑type classification, satellite cell quantification, extracellular matrix analysis, or direct fiber-level agreement metrics. By enabling rigorous quantitative evaluation of routine histology, this approach might facilitate large‑scale retrospective studies and broadens access to morphometric analysis across research and clinical environments.

## Supporting information

S1 Fig IoU comparison between the web-based Cellpose-SAM (CS) and manual segmentation in H&E- and WGA-stained sections. (DOCX)

S2 Table Fiber numbers analyzed with manual correction and exclusion. (DOCX)

S3 File User Tutorial for Myofiber CSA Measurement. User tutorial describing for procedure for myofiber cross‑sectional area measurement.
(DOCX)
