# Supplementary material for: Web-based Cellpose‑SAM segmentation enables quantification of cross-sectional area in formalin-fixed paraffin-embedded skeletal muscle sections stained with hematoxylin and eosin
Source: PLoS One. 2026 Sep 21;21(9):e0358476. doi: 10.1371/journal.pone.0358476 (PMC13592613; doi:10.1371/journal.pone.0358476)
Supplement: Supplementary file 1 [file pone.0358476.s001.docx]

Supplementary Figure 1. IoU comparison between the web-based Cellpose-SAM (CS) and manual segmentation in H&E- and WGA-stained sections

**A	B**

**A.** IoU for manuals-to-manual (M-M, *n*=10 images pairs) and CS-to-manual (CS-M, *n*= 20 image pairs from 10 images) comparisons for H&E-stained sections. **B.** IoU for manuals-to-manual (M-M, *n*=10 images pairs) and CS-to-manual (CS-M, *n*=20 image pairs from 10 images) comparisons for WGA-stained sections.
