# Supplementary material for: Web-based Cellpose‑SAM segmentation enables quantification of cross-sectional area in formalin-fixed paraffin-embedded skeletal muscle sections stained with hematoxylin and eosin
Source: PLoS One. 2026 Sep 21;21(9):e0358476. doi: 10.1371/journal.pone.0358476 (PMC13592613; doi:10.1371/journal.pone.0358476)
Supplement: Supplementary file 2 [file pone.0358476.s002.docx]

Supplementary Table 1. Fiber numbers analyzed with manual correction and exclusion

| Sample | Cellpose-SAM masks fibers (No.) | Initial filtered fibers (No.)^a^ | Manually-added fibers (No.) | Manually-excluded fibers (No.) | Final analyzed fibers (No.) | Manual correction rate (%)^b^ |
| --- | --- | --- | --- | --- | --- | --- |
| Sham #1 | 889 | 884 | 11 | 4 | 891 | 0.79 |
| Sham #2 | 843 | 840 | 0 | 8 | 832 | 0.95 |
| Sham #3 | 963 | 959 | 14 | 2 | 971 | 1.25 |
| SCI #1 | 821 | 816 | 21 | 3 | 834 | 2.21 |
| SCI #2 | 832 | 822 | 42 | 9 | 855 | 4.01 |
| SCI #3 | 927 | 921 | 5 | 10 | 916 | 0.54 |
| SCI #4 | 804 | 798 | 23 | 12 | 809 | 1.38 |

^a^ Initial filtered fibers (No.) indicate the number of fibers remaining from the original Cellpose-SAM segmentation masks after automated filtering via a custom macro (excluding border-touching fibers, area<100 or >4000 μm², and circularity< 0.3).

^b^ Manual correction rate (%) represents the percentage of fibers that required manual intervention (e.g., splitting merged fibers, deleting false positives, or outlining missed fibers) by an investigator prior to downstream morphological analysis, which was obtained by following equation;

Manual correction rate (%) = (Initial filtered fiber No. - Final analyzed fiber No.)/Final analyzed fiber No.
