# Supplementary material for: Web-based Cellpose‑SAM segmentation enables quantification of cross-sectional area in formalin-fixed paraffin-embedded skeletal muscle sections stained with hematoxylin and eosin
Source: PLoS One. 2026 Sep 21;21(9):e0358476. doi: 10.1371/journal.pone.0358476 (PMC13592613; doi:10.1371/journal.pone.0358476)
Supplement: Supplementary file 3 [file pone.0358476.s003.docx]

User Tutorial for Myofiber Cross‑Sectional Area Measurement

1. Install Fiji (ImageJ)

- Go to https://imagej.net/software/fiji/

- Download the Fiji installer for your operating system.

- Install or extract the program.

- Launch Fiji by running the Fiji application.

2. Install MorpholibJ (Required)

- Open Fiji.

- Go to Help → Update...

- Click “Manage Update Sites”.

- Check the box next to “IJPB-plugins”.

- Close the dialog, then click “Apply Changes”.

- Restart Fiji to activate MorpholibJ.

3. Load the Provided Macro

- First, download the macro file myofiber-csa-v1.0.ijm from the GitHub repository: https://github.com/kimjr0/myofiber-csa

- Open Fiji.

- Drag the downloaded macro file (myofiber-csa-v1.0.ijm) into the Fiji window to load it.

- Click Run (triangle button) to execute the macro.

4. Prepare Image Folders

Prepare four folders before running the macro:

- Original images: Raw muscle cross-section images (JPG, TIF, or PNG).

- Masks images: Segmentation masks (downloaded from the Cellpose‑SAM website)

(Important: The filename of each mask must match the original image up to the extension.)

- Filtered masks: Empty folder; macro will save cleaned masks here.

- Result: Empty; macro will store results here.

5. Initial Input Prompts

You will enter:

- Image scale (pixels per micron)

- Length unit (default: um)

Example: If image resolution is 0.5 µm per pixel, scale = 2.0 pixel/um.

6. Folder Selection

A sequence of folder selection windows will appear:

A. Select Original images folder.

B. Select Masks images folder.

C. Select Filtered-masks output folder.

D. Select Analysis Result folder.

7. Filtering Parameters

You will enter:

- Minimum Area

- Maximum Area

- Minimum Circularity

- Manual ROI Review (on/off): These settings help filter out incorrect ROIs, and you can also add new ROIs if needed.

8. Automatic Masks Filtering

For each Masks:

- Masks is opened and border labels removed.

- ROIs are generated using “Label Map to ROIs”.

- Each ROI is measured; ROIs outside the specified range are removed.

- A new filtered Masks is saved.

9. Main Analysis Process

For each original image:

- The original image and its matching filtered Masks are loaded.

- ROIs from the Masks are applied to the original image.

- Initial ROI count is recorded.

- If manual review is enabled:

- ROIs are displayed.

- You can delete or adjust ROIs.

- After pressing OK, updated ROIs regenerate a new filtered Masks file.

10. Final Measurement

- The ROIs are re-measured.

- Results file is saved as CSV.

- Final ROI overlay image is saved as PNG.

- ROI zip file is saved.

- Initial and final ROI counts are added to the summary log.

11. Merged Data File

The macro compiles all area measurements from all images into:

- merged_area_data.csv

12. Summary File

The macro creates:

- analysis_input_summary.txt

Includes:

- Timestamp

- Scale information

- Filtering thresholds

- Manual review status

- ROI counts per image

13. End of Process

The macro prints:

“Analysis Complete”

Processed image count and output folder location.

14. Tips

- Ensure file names between original and mask images match.

- Use manual review to correct segmentation errors.

- Verify ROI overlay images to ensure measurement accuracy.

- Tune area and circularity thresholds depending on tissue type or staining quality.
