# Supplementary material for: Web-based Cellpose‑SAM segmentation enables quantification of cross-sectional area in formalin-fixed paraffin-embedded skeletal muscle sections stained with hematoxylin and eosin
Source: PLoS One. 2026 Sep 21;21(9):e0358476. doi: 10.1371/journal.pone.0358476 (PMC13592613; doi:10.1371/journal.pone.0358476)
Supplement: Supplementary file 4 [file pone.0358476.s004.docx]

**Peer Review Report (6^th^/2/2026)**

*PLOS ONE – Research Article (Manuscript Draft)*

| **Manuscript ID** | PONE-D-25-67815 |
| --- | --- |
| **Title** | Web-based Cellpose-SAM segmentation enables quantification of cross-sectional area in FFPE skeletal muscle sections stained with H&E |
| **Short title** | Web-Based Cellpose-SAM Segmentation for CSA Quantification in FFPE H&E Skeletal Muscle Sections |
| **Article type** | Research Article |
| **Corresponding author** | Jae-Ryong Kim |
| **Reviewer report date** | 2026-02-06 |
| **Reviewer role** | Methodological / computational pathology & quantitative morphometry |

**Overall recommendation: MAJOR REVISION**

**Summary:** The manuscript proposes a practical, web-based workflow that combines Cellpose-SAM segmentation with Fiji/ImageJ post-processing to quantify skeletal muscle myofiber cross-sectional area (CSA) from routine bright-field stains (H&E and toluidine blue) in both FFPE and frozen sections. ***The approach is potentially impactful because it lowers technical barriers and may enable retrospective analysis of archived slides.***

Key limitations to address before the work can be considered technically sound are: (i) inconsistent CSA units in figures (mm² vs µm²), (ii) likely pseudo-replication from fiber-level statistics rather than animal/section-level inference, and (iii) insufficient quantitative validation of segmentation accuracy and reproducibility details for the hosted web tool.

**Editorial checklist (reviewer assessment)**

| **Criterion** | **Assessment** | **Notes** |
| --- | --- | --- |
| Technical soundness of methods | Partially meets | Methods are detailed for staining/animal work, but computational workflow needs additional detail and appropriate statistics. |
| Novelty / contribution | Moderate | Practicality of a browser-based workflow is useful; clearer positioning vs existing H&E-based AI pipelines is needed. |
| Reproducibility | Needs improvement | Hosted web app can change; provide full parameters, versioning, and archived code/data. |
| Statistics | Major concerns | Fiber-level inference likely inflates n; reanalyze at animal-level or with mixed-effects models. |
| Figures & presentation | Needs correction | Correct CSA units on axes; clearly define n (animals vs fibers) in legends. |
| Data & code availability | Partially meets | GitHub macro is helpful; deposit underlying CSA tables and representative images/masks in a public repository. |

**Key Strengths**

- Accessible workflow using a browser-based segmentation interface (no local GPU/Python installation required).
- Feasibility demonstrated on routine FFPE H&E and extended to toluidine blue staining and cryosections.
- Downstream processing is based on open-source Fiji/ImageJ and MorphoLibJ, with a public macro repository.
- Application to sciatic nerve crush captures an expected left-shift in CSA distribution, showing biological sensitivity.

**Major comments**

**1. CSA units are inconsistent between text and figures.**

Results text reports CSA in µm² (e.g., ~1288 µm²), while Figures 2B and 3B label the CSA axis as 'CSA (mm²)' while adjacent distribution panels use µm². Given the plotted ranges (0–5000 or 0–10000), the correct unit is almost certainly µm², not mm².

Please correct all axis labels, legends, and any related text to ensure consistent units throughout; re-check any derived interpretations.

**2. Statistical unit of analysis and pseudo-replication.**

Statistical Analysis states unpaired t-tests and KS tests; Results use large fiber counts (hundreds) as n. **Issue:** Fibers are nested within animals/sections, so treating each fiber as an independent observation inflates the effective sample size and can produce spuriously small p-values.

Reanalyze using animal-level inference (e.g., per-animal mean/median CSA and compare groups with n=number of mice) and/or mixed-effects models with random intercepts for animal (and section/tile if relevant). For distributional comparisons, use animal-level bootstrapping or hierarchical methods. Report effect sizes with 95% confidence intervals and clearly define the biological replicate.

**3. Add quantitative validation of segmentation accuracy/agreement.**

Feasibility and stain concordance are shown, but accuracy is not quantified against a reference standard.

On a representative subset of images (multiple animals, stains, tissue types), compare Cellpose-SAM outputs to (i) manual ground-truth annotations and/or (ii) immunofluorescence-based basal lamina segmentation. Report Dice/IoU (or similar), split/merge error rates, and downstream CSA agreement (Bland–Altman bias/limits, correlation). Also report how many fibers required manual correction/exclusion.

**4. Reproducibility risks from reliance on a hosted web app; improve parameter reporting and archiving.**

Methods indicate use of a Hugging Face Cellpose-SAM interface and 'default settings'.

**Issue:** Hosted services and defaults can change over time; 'default' is not reproducible unless fully specified.

Provide complete parameter values (as shown in the interface), exact model identifiers, and any version/commit information available. In addition to GitHub, create an archived release (e.g., Zenodo DOI) of the macro and documentation. Consider providing a local/scriptable alternative (Python command line or container) for long-term reproducibility.

**5. Whole-slide image handling is unclear given file-size limits; specify tiling/downsampling and aggregation.**

Methods note a 10 MB upload limit. Whole-slide 40× images usually exceed this, yet Results imply whole-section analysis.

Describe the exact export strategy (downsampling factor, tile size/overlap, number of tiles per section, how boundary fibers were handled to avoid double counting). If downsampled images were used, quantify the impact on CSA bias and provide recommended minimal resolution.

**6. Pixel calibration and measurement integrity must be explicitly documented.**

CSA computation requires correct pixel-to-micron calibration in Fiji/ImageJ.

Specify how scale calibration was set (scanner metadata, known calibration, scale-bar reference) and ensure the macro enforces calibration consistently across all images. Include a simple calibration QC step and report tolerance.

**7. Clarify sciatic nerve crush design and control structure; address potential confounding by laterality.**

Methods describe bilateral harvest and assigning left vs right muscles to frozen vs FFPE processing. **Issue:** If injury is unilateral, laterality may confound comparisons; if bilateral, this must be stated.

State clearly whether crush was unilateral or bilateral, which side(s) were injured, whether contralateral controls were used, and whether analysts were blinded during ROI curation and measurement.

**Summary table:**

| **Major point** | **What to change** | **Where to report it** | **Evidence to provide** |
| --- | --- | --- | --- |
| Units | Fix CSA axis units (Figures 2B and 3B) to µm²; re-check labels and text | Figures 2B and 3B; figure legends; Results | Updated plots and legend text |
| Statistics | Use animal-level n or mixed-effects; report effect sizes/CI | Statistical Analysis; Results; legends | Revised tables/plots; model specification |
| Accuracy validation | Compare to ground truth or IF-based segmentation; report Dice/IoU and CSA agreement | New Results subsection + supplement | Annotated dataset + metrics + example overlays |
| Reproducibility | List full parameters; version/commit; archive code/data (DOI) | Methods; Data availability | Parameter table + DOI link + repository contents |
| Whole-slide handling | Describe tiling/downsampling and aggregation | Methods; Supplement | Flowchart or parameter table; example tile grid |
| Calibration | Describe calibration method and QC | Methods; Supplement | Calibration check results |
| Injury design | Clarify laterality; controls; blinding | Methods; Results | Diagram or explicit text; ARRIVE items |

**Minor comments**

1. Grammar/wording: in the Introduction, 'H&E staining remains ubiquitous in histopathology and are compatible…' should be 'is compatible…'. Review for similar issues.
2. Clarify which muscles were analyzed as whole sections (soleus only, or additional muscles listed in Methods). If additional, provide at least one example or summarize in Supplement.
3. Provide explicit criteria/thresholds for morphological filtering (min/max area, circularity/solidity, artifact exclusion) or add a Supplementary Table of parameter values.
4. Report how many fibers were excluded during QC (boundary fibers, debris, tears) and present a flow diagram (images → masks → filtered ROIs → final fibers).
5. Processing time: specify image dimensions and typical tile count; web app latency depends on image size and server load.
6. Data availability: deposit raw CSA tables (per fiber and per animal), representative input images, and masks in a public repository compliant with PLOS policy.
7. References: the SAM citation is incomplete; provide full citation (e.g., arXiv identifier).
8. Statistical reporting: include exact p-values where feasible and define which summary statistic is shown in violin plots (median/IQR vs mean/SD).
9. Define 'default settings' precisely for Cellpose-SAM (including any resizing/normalization performed by the interface).
10. Terminology: use consistent ASCII hyphenation (Cellpose-SAM) throughout.

**Critical section notes:**

**Title/Abstract**

Clear and informative. After reanalysis, add one sentence specifying the number of animals and the statistical unit of inference.

**Introduction**

Well-motivated. Consider explicitly contrasting with existing H&E-based AI approaches in skeletal muscle pathology (what is distinct about a fully web-based pipeline).

**Materials & Methods**

Animal and staining methods are detailed. The computational workflow needs a reproducible parameter table and explicit tiling/calibration steps. Also clarify blinding and define the experimental unit.

**Results**

Logical sequence. Correct figure units and reanalyze statistics. Add quantitative segmentation accuracy and (ideally) inter-rater reliability for ROI curation.

**Discussion**

Balanced. Add limitations regarding reliance on a hosted web service, generalization across staining variability, and performance in severe pathology/regenerating areas.

**Figures**

Workflow figure is helpful. Ensure all axes/units are correct; define n and summary statistics used in plots.

**Ethics & reporting**

Ethics statement and humane endpoints are included. Consider adding ARRIVE-style items (sample-size rationale, blinding, inclusion/exclusion criteria).
