# Supplementary material for: Web-based Cellpose‑SAM segmentation enables quantification of cross-sectional area in formalin-fixed paraffin-embedded skeletal muscle sections stained with hematoxylin and eosin
Source: PLoS One. 2026 Sep 21;21(9):e0358476. doi: 10.1371/journal.pone.0358476 (PMC13592613; doi:10.1371/journal.pone.0358476)
Supplement: Supplementary file 5 [file pone.0358476.s005.docx]

**Authors’ Responses to Reviewers’ Comments**

**Major comments**

**1. CSA units are inconsistent between text and figures.**

Results text reports CSA in µm² (e.g., ~1288 µm²), while Figures 2B and 3B label the CSA axis as 'CSA (mm²)' while adjacent distribution panels use µm². Given the plotted ranges (0–5000 or 0–10000), the correct unit is almost certainly µm², not mm².

Please correct all axis labels, legends, and any related text to ensure consistent units throughout; re-check any derived interpretations.

(Response)

We sincerely thank the reviewer for catching this typographical error. The reviewer is absolutely correct; the proper unit for myofiber CSA is μm^2^, not mm^2^. We have carefully corrected the y-axis labels in Figs. 4B and 5B in the revised manuscript. Furthermore, as requested, we have double-checked all derived data and interpretations. We confirm that this was strictly a visual labeling error; all underlying CSA measurements, statistical outcomes, and interpretations were already correctly based on μm^2^ and remain entirely unaffected.

**2. Statistical unit of analysis and pseudo-replication**

Statistical Analysis states unpaired t-tests and KS tests; Results use large fiber counts (hundreds) as n.

**Issue:** Fibers are nested within animals/sections, so treating each fiber as an independent observation inflates the effective sample size and can produce spuriously small p-values.

Reanalyze using animal-level inference (e.g., per-animal mean/median CSA and compare groups with *n*=number of mice) and/or mixed-effects models with random intercepts for animal (and section/tile if relevant). For distributional comparisons, use animal-level bootstrapping or hierarchical methods. Report effect sizes with 95% confidence intervals and clearly define the biological replicate.

(Response)

We thank the reviewer for highlighting the issue of pseudo-replication. We agree that individual fibers are nested within sections/animals and therefore are not independent biological replicates; treating fibers as independent observations can inflate the effective sample size and yield spuriously small *p*-values.

Accordingly, we reanalyzed CSA using animal-level inference, defining the biological replicate as the individual mouse (Sham, *n*=3; SCI, *n*=4). Given the small number of animals, we focused on conservative animal-level inference (per-animal summaries) as the primary analysis to directly reflect the biological replication structure. For each animal, we pooled all segmented fibers to compute a single per-animal mean and per-animal median CSA, and performed group comparisons using *n*=number of animals. The updated figures show fiber distributions for each animal (Fig. 6E) and the per-animal summary (mean or median) values used for inference (Fig. 6F and 6G).

Using this animal-level approach, the SCI group still exhibited a significant reduction compared to the sham group (Fig. 6E-6H). We now report effect sizes with 95% confidence intervals (CIs) based on these animal-level summaries. The revised Results section can be found on Pages 17-18, Lines 393–397.

Finally, the relative frequency distributions derived from multiple samples were re-evaluated using the Kolmogorov-Smirnov test, and the exact *p*-value was included in Fig. 6H.

**3. Add quantitative validation of segmentation accuracy/agreement.**

Feasibility and stain concordance are shown, but accuracy is not quantified against a reference standard.

On a representative subset of images (multiple animals, stains, tissue types), compare Cellpose-SAM outputs to (i) manual ground-truth annotations and/or (ii) immunofluorescence-based basal lamina segmentation. Report Dice/IoU (or similar), split/merge error rates, and downstream CSA agreement (Bland–Altman bias/limits, correlation). Also report how many fibers required manual correction/exclusion.

(Response)

We sincerely thank the reviewer for pointing out the critical need for quantitative validation against a reference standard. We fully agree with this assessment. To address this, we have performed the requested quantitative analyses and updated the manuscript as detailed below.

i) Comparisons Cellpose-SAM outputs to manual ground-truth annotations and/or immunofluorescence-based basal lamina segmentation. Report Dice/IoU and assessment of split/merge errors.

1) Assessment of split/merge errors via Dice coefficient

To functionally address split or merge errors, we utilized the Dice coefficient, which robustly captures these spatial deviations. Rather than reporting an isolated error rate, we directly compared the accuracy of Cellpose-SAM (CS) outputs (CS-to-Manual, CS-M) against inherent human variability (Manual-to-Manual, M-M baseline).

2) Comparison to manual ground-truth (H&E analysis)

First, we validated the automated Cellpose-SAM (CS) outputs on routine H&E-stained images by comparing them against manual (M) tracings. To establish a rigorous reference standard, manual annotations for representative images (*n*=10) were independently performed by two investigators (M1 and M2) to define the M-M baseline. When comparing the automated CS outputs to these annotations, there was no significant difference in the Dice coefficient between the M-M baseline and the CS-M group (*p*=0.1971) in the revised Figs. 3A and 3B. This concisely confirms that any minor deviations generated by Cellpose-SAM (such as split/merge tendencies) strictly fall within the normal range of human observer variability, achieving human-level accuracy.

3) Comparison to ECM/basal lamina segmentation (WGA analysis)

Second, addressing the reviewer’s point regarding immunofluorescence-based basal lamina segmentation, traditional markers (e.g., laminin or collagen IV) are often restricted to cryosections due to epitope masking during FFPE procedures. Therefore, to establish a robust reference standard for true myofiber boundaries in FFPE tissues, we utilized WGA staining, a lectin that reliably binds to sarcolemmal glycoproteins and the extracellular matrix (PMID: 21181705). Applying the exact same validation design (M-M vs. CS-M) to WGA-stained images, the automated CS segmentation similarly showed no significant difference compared to manual tracing (*p*=0.1179; Fig. 3C). Furthermore, the choice of staining (H&E vs. WGA) did not significantly affect the mean cross-sectional area (CSA) or their relative frequency distributions (Fig. 3D and 3E).

iii) Report downstream CSA agreement (Bland–Altman bias/limits, correlation)

Initially, we tried to perform a fiber-to-fiber correlation and Bland-Altman analysis as suggested. However, directly matching hundreds of individual fibers 1:1 across different modalities (e.g., consecutive sections of H&E and WGA) introduces unavoidable spatial registration artifacts. Therefore, we utilized an alternative, highly comprehensive approach to robustly validate the downstream morphometric agreement:

- Mean CSA: The automated pipeline showed no significant difference in the mean CSA between H&E and WGA images (*p*=0.0848, Fig. 3D).
- Fiber size distribution: More importantly, we evaluated the fiber size frequency distribution across the entire pooled dataset of individual fibers. This method is highly sensitive to segmentation biases; for instance, split errors would unnaturally skew the curve to the left, while merge errors would skew it to the right. As depicted in Fig. 3E, the frequency curves of H&E overlapped with the those of WGA. Statistical analysis confirmed no significant difference between the two distribution patterns (*p*=0.9994).

We have fully detailed these validation procedures in the revised Results section (Pages 14-15, Lines 315-346) and Fig. 3.

iv) Report how many fibers required manual correction/exclusion:

To provide full transparency regarding the post-processing steps as requested, we have quantified the exact number of fibers requiring manual correction or exclusion after the initial segmentation by Cellpose-SAM and subsequent automated filtering via our custom macro. These manual intervention metrics, evaluated on the 10 H&E and WGA-stained images used for manual ground-truth annotations in Fig. 3, are detailed in the Table. Overall, the Table highlights that manual intervention is rarely needed after our automated segmentation. With manual modification rates remaining exceptionally low (0-1.4% for H&E and 0-5.3% for WGA), these metrics decisively prove the high precision and reliability of our Cellpose-SAM and macro-driven workflow.

| Staining | Image number | Original ROIs | Manual modification ROIs | Final ROIs | Modification rate (%) |
| --- | --- | --- | --- | --- | --- |
| H&E | 1 | 47 | 0 | 47 | 0 |
|  | 2 | 57 | 0 | 57 | 0 |
|  | 3 | 72 | 0 | 72 | 0 |
|  | 4 | 64 | 0 | 64 | 0 |
|  | 5 | 65 | 0 | 65 | 0 |
|  | 6 | 65 | 0 | 65 | 0 |
|  | 7 | 70 | 1 | 69 | 1.4 |
|  | 8 | 70 | 1 | 69 | 1.4 |
|  | 9 | 62 | 0 | 62 | 0 |
|  | 10 | 61 | 0 | 61 | 0 |
| WGA | 1 | 46 | 0 | 46 | 0 |
|  | 2 | 54 | 0 | 54 | 0 |
|  | 3 | 69 | 0 | 69 | 0 |
|  | 4 | 62 | 2 | 60 | 3.2 |
|  | 5 | 70 | 2 | 68 | 2.7 |
|  | 6 | 70 | 0 | 70 | 0 |
|  | 7 | 72 | 4 | 68 | 5.3 |
|  | 8 | 69 | 0 | 69 | 0 |
|  | 9 | 61 | 1 | 60 | 1.6 |
|  | 10 | 61 | 1 | 60 | 1.6 |

Table. Evaluation of manual interventions required post-Cellpose-SAM and macro filtering.

In addition, we presented manual correction rate in whole soleus muscle cross-sections obtained from mice (Sham, *n*=3 and SCI, *n*=4). As detailed in the newly added Supplementary Table 1, the need for manual intervention was minimal. Across all analyzed whole-muscle images, manually added fibers ranged from only 0 to 42, and manually excluded fibers ranged from 2 to 12 per image. Notably, the overall manual correction rate remained exceedingly low, ranging from 0.54% to 4.01%. These quantitative results strongly demonstrate the high accuracy and out-of-the-box robustness of our web-based Cellpose-SAM segmentation pipeline prior to any human intervention.

In conclusion, these comprehensive validations confirm that our web-based Cellpose-SAM pipeline captures the true morphometric profile without introducing systematic sizing biases. Ultimately, it establishes that routine H&E staining, driven by our web-based Cellpose-SAM workflow, serves as a highly effective and accessible substitute for traditional fluorescence-based segmentation*.*

**4. Reproducibility risks from reliance on a hosted web app; improve parameter reporting and archiving.**

Methods indicate use of a Hugging Face Cellpose-SAM interface and 'default settings'.

**Issue:** Hosted services and defaults can change over time; 'default' is not reproducible unless fully specified. Provide complete parameter values (as shown in the interface), exact model identifiers, and any version/commit information available. In addition to GitHub, create an archived release (e.g., Zenodo DOI) of the macro and documentation. Consider providing a local/scriptable alternative (Python command line or container) for long-term reproducibility.

(Response)

We sincerely thank the reviewer for raising this critical point regarding long-term reproducibility and open science. We fully agree that relying solely on a third-party hosted web interface and stating "default settings" is insufficient, as web applications and defaults are subject to change over time. To ensure robust and permanent reproducibility, we have addressed all reviewer’s recommendations and updated the Methods section accordingly:

i) Complete parameter values and exact model identifiers
We have revised the Methods section to explicitly detail the exact parameters and model identifiers used in the version 1.2.0 interface. Rather than older segmentation models, the web application utilizes the unified Cellpose-SAM model (identifier: *cpsam*), which is built upon the ViT-L (Vision Transformer-Large) backbone architecture from the Segment Anything Model. The default segmentation parameters were applied strictly without any modifications, and they are now fully specified in the manuscript as follows: max resize = 1000, max iter = 250, flow thresh = 0.4, and cellprob thresh = 0. These details are stated on Page 9, Lines 197-202, and in Fig. 1B.

ii) Local/scriptable alternative for long-term reproducibility
We completely agree with the potential risks of hosted services. To guarantee long-term reproducibility regardless of the Hugging Face interface's status, we have developed a local, scriptable alternative Python script (run_cellpose_local.py). This allows users to easily execute the exact Cellpose-SAM pipeline locally on their own machines using the identical parameters reported in our study.

iii) Archival release via Zenodo
To ensure the immutability and permanent availability of our complete workflow, we have created an archived release containing the local Python script, our custom Fiji/ImageJ macro, detailed user documentation, representative images, and raw data files. This repository is now permanently archived and publicly accessible via Zenodo (DOI: 10.5281/zenodo.19447533).

**5. Whole-slide image handling is unclear given file-size limits; specify tiling/downsampling and aggregation.**

Methods note a 10 MB upload limit. Whole-slide 40× images usually exceed this, yet Results imply whole-section analysis. Describe the exact export strategy (downsampling factor, tile size/overlap, number of tiles per section, how boundary fibers were handled to avoid double counting). If downsampled images were used, quantify the impact on CSA bias and provide recommended minimal resolution.

(Response)

We deeply appreciate the reviewer’s careful attention to whole-slide image (WSI) handling and the potential for resolution-dependent CSA bias. We would like to clarify our exact export strategy: while we did apply a moderate downsampling factor, our analysis did not involve any image splitting or tiling.

i) Export strategy (Single image with a downsampling factor of 4)
Unlike massive human tissue sections, the entire cross-section of a mouse soleus muscle is anatomically small. To export the whole section as a single, contiguous image while strictly adhering to the platform's file-size limits, we applied a downsampling factor of 4 during export. Because the entire section was processed as a single image, there were no grid tiles to merge, and consequently, absolutely no risk of double-counting fibers. Furthermore, any incomplete boundary fibers touching the absolute extreme edges of the single whole-section image were automatically excluded using the 'Remove Border Labels' function in MorphoLibJ.

ii) Optimization of downsampling and CSA bias
To address the reviewer's valid concern regarding downsampling-induced CSA bias, we have added our systematic multi-resolution optimization data in Fig. 2. We systematically exported WSI resolutions from D1 (4.00 pixel/μm) down to D16 (0.25 pixel/μm) via QuPath and analyzed them. We purposefully selected D4 (1.00 pixel/μm) as the operational reference. This specific downsampling factor effectively suppressed intracellular textural noise (which impairs deep-learning segmentation) while retaining robust boundary fidelity. Crucially, our quantitative validation confirmed that the D4 resolution (yielding final WSI resolutions generally ranging from 0.8 to 1.0 pixel/μm) introduced no significant morphometric bias in CSA calculations compared to the native resolution. To ensure absolute transparency, the exact spatial resolution for each analyzed image has been explicitly detailed in the figure legends of Fig. 2A. To answer reviewer’s final point, we recommend a minimal spatial resolution of 0.8 pixel/μm (ideal range: 0.8-2.0 pixel/μm) for the web-based Cellpose-SAM workflow.

These details are stated on Pages 13-14, Lines 292-313, and in Fig. 2.

**6. Pixel calibration and measurement integrity must be explicitly documented.**

CSA computation requires correct pixel-to-micron calibration in Fiji/ImageJ.

Specify how scale calibration was set (scanner metadata, known calibration, scale-bar reference) and ensure the macro enforces calibration consistently across all images. Include a simple calibration QC step and report tolerance.

(Response)

We deeply appreciate the reviewer’s highly constructive feedback regarding pixel calibration. We completely agree that these technical parameters are crucial for measurement integrity. We have extensively revised the Methods section (under the new subheadings “Image preparation and downsampling optimization” on Pages 8-9, Lines 166-192 and “Pixel (px) calibration, morphological filtering, and CSA measurement” on Page 10, Lines 206-226) to explicitly address the reviewer’s points.

We strictly documented our pixel-to-micron calibration protocol to guarantee measurement integrity:

- Calibration source: The exact spatial calibration was mathematically derived directly from the native optical metadata of the Magscanner KF-PRP-005 slide scanner (KFBIO Technology for Health, Yuyao, China) via QuPath during the downsampled export.
- Macro enforcement: To eliminate any risk of metadata loss during file conversion, this specific spatial calibration was hardcoded into our custom Fiji/ImageJ batch-processing macro. The macro executes the *Set Scale* command uniformly on every image before any morphological measurements occur.
- QC step & tolerance: Prior to batch processing, we implemented a manual quality control (QC) step. We exported images containing a scanner-generated reference scale bar and manually measured its length in Fiji/ImageJ after applying our macro's scale. This QC step confirmed that the calibration was perfectly enforced, yielding a measurement tolerance of less than 1%.

We believe these comprehensive methodological clarifications strongly reinforce the technical validity and reproducibility of our automated CSA quantification pipeline.

**7. Clarify sciatic nerve crush design and control structure; address potential confounding by laterality.**

Methods describe bilateral harvest and assigning left vs right muscles to frozen vs FFPE processing. **Issue:** If injury is unilateral, laterality may confound comparisons; if bilateral, this must be stated.

State clearly whether crush was unilateral or bilateral, which side(s) were injured, whether contralateral controls were used, and whether analysts were blinded during ROI curation and measurement.

(Response)
We sincerely thank the reviewer for raising these critical points regarding the experimental design. We apologize if the original manuscript lacked explicit clarity on these details. We have comprehensively revised the Methods section to clarify the bilateral nature of our model, the control structure (Page 5, Line 98-108), and our rigorous blinding process (Page 6, Line 129-131; Page 10, Lines 223-226).

i) Bilateral injury design and true control structure
To completely eliminate the potential laterality confound pointed out by the reviewer, the sciatic nerve crush injury was performed bilaterally (on both the left and right hindlimbs). Because both hindlimbs were injured, we did not use contralateral uninjured legs as controls. Instead, true biological control samples were obtained from separate sham-operated mice, which underwent identical bilateral surgical procedures (including nerve exposure) but without the actual crush injury.

ii) Justification for left vs. right assignment
Because both hindlimbs within a single injured mouse experienced the exact same pathophysiological condition, assigning the left-side muscles to cryopreservation and the right-side muscles to FFPE processing was a highly controlled strategy. This specific bilateral design allowed for a direct, within-subject comparison of the histological processing methods (Frozen vs. FFPE) while inherently controlling for inter-subject biological variation.

iii) Blinding during image acquisition and ROI curation
As requested, we have clarified our blinding and measurement protocols. Analysts were strictly blinded to the sample groups and injury status during tissue processing and whole-slide image acquisition. Furthermore, regarding ROI curation and measurement: while the initial myofiber segmentation was efficiently automated using the Cellpose-SAM model, the downstream ROI generation and final extraction of cross-sectional area (CSA) data were performed using a custom Fiji/ImageJ macro. Crucially, during this macro-assisted process, a strictly blinded investigator rigorously inspected all masks and manually corrected any improperly segmented boundaries (e.g., falsely merged or split fibers). This combined approach (Cellpose-SAM initial segmentation followed by blinded manual curation) ensured maximal morphometric accuracy while completely preventing subjective human bias during measurement.

**Minor comments**

1. Grammar/wording: in the Introduction, 'H&E staining remains ubiquitous in histopathology and are compatible…' should be 'is compatible…'. Review for similar issues.

(Response)
We thank the reviewer for careful reading and for catching this grammatical error. We have corrected the phrase to 'is compatible' in the Introduction (Page 3, Line 53). Furthermore, we have carefully reviewed the entire manuscript and corrected other minor grammatical issues and typos to improve the overall readability.

2. Clarify which muscles were analyzed as whole sections (soleus only, or additional muscles listed in Methods). If additional, provide at least one example or summarize in Supplement.

(Response)
We thank the reviewer for pointing out this discrepancy between the initially harvested tissues and the presented data.

As listed in the Methods, multiple hindlimb muscles were harvested and initially digitized via the slide scanner. However, following a comprehensive quality-control review in QuPath, only the soleus muscle was ultimately selected for the automated whole-slide morphometric analysis presented in this study. The soleus muscle was specifically chosen as the representative model for our automated analysis pipeline because its relatively small anatomical size allows for optimal complete cross-sectional evaluation without artifactual fragmentation, and its vulnerability to sciatic nerve crush injury is well-documented (PMID: [17938279](https://pubmed.ncbi.nlm.nih.gov/17938279/)). To avoid any confusion, we have explicitly clarified this sequential selection process (initial harvest and scanning of all muscles → final selection of the soleus) at the beginning of the "Image preparation and downsampling optimization" subsection in the revised Methods (Page 8, Lines 170-175).

3. Provide explicit criteria/thresholds for morphological filtering (min/max area, circularity/solidity, artifact exclusion) or add a Supplementary Table of parameter values.

(Response)

As requested, we have explicitly detailed the morphological filtering criteria in the revised Methods section (Page 10, Lines 214-219) and Fig. 1C.

The specific thresholds programmed into our Fiji/ImageJ macro to exclude non-myofiber artifacts include: (1) a size range of 100 μm^2^ to a variable upper limit (4000, 6000, or 10000 μm^2^, depending on the image), (2) a circularity threshold of ≥ 0.3, and (3) the exclusion of incomplete border-touching fibers using the MorphoLibJ plugin. We believe these clear descriptions fully address the reviewer's request regarding filtering parameters.

4. Report how many fibers were excluded during QC (boundary fibers, debris, tears) and present a flow diagram (images → masks → filtered ROIs → final fibers).

(Response)

i) Quantification of excluded and corrected fibers

To guarantee high morphometric accuracy at the image peripheries, we employed the "Remove Border Labels" function via the MorphoLibJ plugin within our custom macro. This safely excludes any partially cut myofibers touching the borders before calculating the CSA. As illustrated in Fig. 3A using the H&E and WGA segmentation datasets, our macro efficiently detects and removes these boundary-intersecting fibers, confirming the strict quality control of our pipeline.

To address the exact number of fibers excluded during our QC process, we have provided Supplementary Table 1. Please see our detailed response provided under Major Comment #3-iii) for further information regarding this validation.

Regarding tissue tears, regions with obvious tears or folding were preemptively avoided during the initial whole-slide screening in QuPath; therefore, they were excluded prior to the initial segmentation.

ii) Presentation of the flow diagram

To fulfill the reviewer’s request for a visual flow diagram (images → masks → filtered ROIs → final fibers), we have specifically designed Fig. 1 in the revised manuscript to serve exactly this purpose.

5. Processing time: specify image dimensions and typical tile count; web app latency depends on image size and server load.

(Response)

We thank the reviewer for this insightful comment. We had reported the average processing time as 7.5±2.1 seconds per image. In addition, we now specify that the dimensions of these evaluated images as pixels in the legends of Figs. 2A, 3A, 4A, 5A, and 6D.

Regarding the tile count, we inputted the whole image of the soleus muscle directly into the Cellpose-SAM web application without any manual cropping. As the web application processes the uploaded image automatically, any tiling is managed internally by the system, so a specific user-defined tile count cannot be provided.

Furthermore, directly following our reported processing time, we have explicitly added a statement acknowledging that this processing speed represents an observed average under our specific experimental conditions. We clearly note that actual application latency will naturally fluctuate depending on specific image sizes, local internet bandwidth, and concurrent GPU server load at the time of analysis.

6. Data availability: deposit raw CSA tables (per fiber and per animal), representative input images, and masks in a public repository compliant with PLOS policy.

(Response)

We fully agree with the reviewer and strictly adhere to the PLOS data availability policy. We recognize that sharing our raw datasets and representative images is essential for ensuring methodological transparency and allowing the community to reproduce our automated analytical pipeline.

Accordingly, we have deposited the comprehensive dataset into Zenodo (DOI: 10.5281/zenodo.19447533). To directly address the reviewer's request, the deposited public dataset explicitly includes: Raw CSA measurement tables (detailed both per individual fiber and aggregated per animal), Representative input images used for CSA evaluation, The corresponding mask files generated via Cellpose-SAM and filtered through our Fiji/ImageJ macro.

Furthermore, we have updated the "Data availability statement" during the manuscript submission process to reflect this permanent public repository link, fully complying with PLOS guidelines.

7. References: the SAM citation is incomplete; provide full citation (e.g., arXiv identifier).

(Response)

We have updated the reference list to provide the full citation for the SAM paper, including the arXiv identifier, as requested. The revised reference (Reference No. 11) can be found on Page 22, Lines 511-514 of the revised manuscript. Additionally, all newly added references have been clearly highlighted in red within the updated reference list.

8. Statistical reporting: include exact p-values where feasible and define which summary statistic is shown in violin plots (median/IQR vs mean/SD).

(Response)

We deeply appreciate the reviewer's careful attention to our statistical reporting, which helps improve the precision of our manuscript.

First, regarding the exact *p*-values, we have thoroughly reviewed the manuscript and ensured that exact *p*-values are explicitly provided on the Figures.

Second, regarding the summary statistics in the violin plots, we have updated the corresponding figure legends to prevent any reader confusion. We have explicitly defined that the internal lines represent the median and quartiles (specifically, the solid green line represents the median, and the dashed green lines represent the quartiles) in the legends of Figs. 3B, 3C, 4B, 5B, and 6E.

9. Define 'default settings' precisely for Cellpose-SAM (including any resizing/normalization performed by the interface).

(Response)
We have thoroughly updated the Methods section to explicitly define what the "default settings" entail in the Cellpose-SAM web application. Furthermore, these exact parameters have been visually summarized in the flow diagram of Fig. 1B.

Specifically, the default parameters applied were max resize = 1000 (which rescales the maximum dimension of the uploaded image to 1000 pixels while preserving the aspect ratio to optimize web server processing), max iter = 250 (the maximum number of iterations for the network), flow threshold = 0.4, and cellprob threshold = 0.

Regarding normalization, we also clarified in the manuscript that the interface automatically utilizes Cellpose's standard built-in normalization, which scales the input image pixel intensities to a 0-1 range based on the 1st and 99th percentiles before feeding them into the network.

10. Terminology: use consistent ASCII hyphenation (Cellpose-SAM) throughout.

(Response)

We have thoroughly reviewed the entire revised manuscript, including the main text, figure legends, tables, and supplementary materials. We corrected the standard ASCII hyphenation, "Cellpose-SAM" and others.
