# Supplementary material for: Web-based Cellpose‑SAM segmentation enables quantification of cross-sectional area in formalin-fixed paraffin-embedded skeletal muscle sections stained with hematoxylin and eosin
Source: PLoS One. 2026 Sep 21;21(9):e0358476. doi: 10.1371/journal.pone.0358476 (PMC13592613; doi:10.1371/journal.pone.0358476)
Supplement: Supplementary file 6 [file pone.0358476.s006.docx]

**Re-Review Report**

| Journal | PLOS ONE |
| --- | --- |
| **Manuscript ID** | PONE-D-25-67815R1 |
| **Article type** | Research Article |
| **Title** | Web-based Cellpose-SAM segmentation enables quantification of cross-sectional area in FFPE skeletal muscle sections stained with H&E |
| **Re-review focus** | Assessment of whether the authors adequately addressed the previous major and minor comments |

**Overall recommendation: ACCEPT AFTER MINOR REVISION / FINAL TECHNICAL CHECK**

The authors have addressed the major methodological and reporting concerns substantially. No further major experimental work appears necessary. Remaining issues are mainly clarity, statistical framing, transparency of validation metrics, and moderation of overstated claims.

# 1. Executive summary

The revised manuscript is **substantially improved** compared with the version that received major revision. The authors have corrected the obvious unit error, reanalyzed the key injury comparison using animal-level inference, added quantitative segmentation validation, strengthened reproducibility through parameter reporting and an archived release, clarified whole-slide export/downsampling, documented calibration, and resolved the laterality/blinding ambiguity in the sciatic nerve crush model.

From a research process perspective, the revision demonstrates good faith engagement with the reviewer comments and materially strengthens the credibility, reproducibility, and interpretability of the study.

The main remaining concern is not the feasibility of the method but the precision with which the authors frame inference. Fiber-level distributions are valuable descriptively, but biological inference should remain anchored at the animal or image/section level. The revised manuscript should ensure that all figures, legends, and statistical statements clearly distinguish descriptive fiber-level distributions from inferential comparisons based on biological replicates.

# 2. Decision rationale

| Domain | Previous concern | Current status | Residual action required |
| --- | --- | --- | --- |
| Technical soundness | CSA unit errors, unclear WSI handling, calibration not explicit. | Substantially improved. | Final visual check of all figures and legends. |
| Statistical validity | Pseudo-replication from treating fibers as independent observations. | Improved for SCI/sham comparison through animal-level summaries. | Clarify statistical unit across all analyses and avoid inferential claims from pooled fiber counts. |
| Segmentation validation | Feasibility shown but limited accuracy metrics. | Improved through manual annotation, WGA comparison, Dice-based validation, and manual correction rates. | Report actual Dice/IoU values and uncertainty where possible; avoid overclaiming. |
| Reproducibility | Hosted web app and “default settings” not reproducible. | Strongly improved through exact parameters, local script, and Zenodo archive. | Verify archive content and license/accessibility. |
| Experimental design | Laterality and blinding unclear. | Adequately clarified as bilateral injury with separate sham controls and blinded curation. | Use cautious wording: blinding reduces, but does not completely eliminate, bias. |

# 3. Point-by-point assessment of authors' responses

| No. | Reviewer comment | Authors' response / revision | Re-review assessment | Verdict |
| --- | --- | --- | --- | --- |
| 1 | CSA units inconsistent between text and figures. | Corrected CSA axis labels from mm2 to um2 and stated that underlying measurements and interpretations were unaffected. | This is satisfactory, provided the revised figures and legends indeed use um2 consistently. The issue appears to have been a labelling error rather than an analytical error. | Addressed |
| 2 | Pseudo-replication / statistical unit of analysis. | Reanalyzed the key sham vs SCI comparison at animal level, defining the mouse as the biological replicate (Sham n=3; SCI n=4), using per-animal mean/median CSA and effect sizes with 95% CIs. | Substantially improved. However, the revised text should still make very clear where fiber counts are descriptive and where animal-level values are inferential. Any KS tests on pooled fibers or multiple samples should not be presented as independent biological inference unless hierarchical or animal-level methods are used. | Partly addressed; minor revision |
| 3 | Add quantitative segmentation accuracy/agreement validation. | Added H&E manual-ground-truth validation, WGA boundary validation, Dice-based comparison against manual-manual variability, and manual correction/exclusion counts. | This is a major improvement. The validation is credible for a practical methods paper. Remaining limitations: actual Dice/IoU values and confidence intervals should be clearly reported; split/merge errors are inferred rather than directly counted; Bland-Altman/correlation were not performed, though the authors provide a plausible registration-based explanation. | Substantially addressed; minor clarification |
| 4 | Improve reproducibility of hosted web app workflow. | Provided interface version/model identifier, exact default parameters, local Python alternative, and Zenodo archive DOI with script, macro, documentation, representative images, and data. | Satisfactory and strong. The archive should be checked for completeness, public accessibility, persistent DOI resolution, and inclusion of license/readme files. | Addressed |
| 5 | Clarify whole-slide handling, downsampling, tiling, aggregation. | Clarified single-image export of mouse soleus at D4/downsampling factor 4, no user tiling, border-label removal, and recommended spatial resolution. | Adequately addressed for the specific mouse soleus use case. Authors should clarify that recommendations may not generalize automatically to larger human sections or other muscles that require tiling. | Addressed with caveat |
| 6 | Document pixel calibration and measurement integrity. | Added scanner metadata-based calibration, hardcoded Fiji/ImageJ Set Scale enforcement, reference scale-bar QC, and tolerance <1%. | Satisfactory. The manuscript should state where the calibration QC data are provided or add a brief supplementary confirmation table. | Addressed |
| 7 | Clarify sciatic nerve crush laterality, controls, and blinding. | Clarified bilateral injury, separate sham-operated controls, left/right assignment for tissue processing, and blinded acquisition/ROI curation. | Adequately addressed. The wording should be moderated: blinding reduces subjective bias but does not “completely prevent” all bias. | Addressed; wording edit |

# 4. Minor comments and reporting issues

The authors have mostly addressed the minor comments. The remaining edits are editorial and transparency-related rather than substantive barriers to publication.

| Area | Author action | Re-reviewer judgment | Need before acceptance |
| --- | --- | --- | --- |
| Grammar and terminology | Corrected “is compatible” and standardized Cellpose-SAM hyphenation. | Acceptable. | Routine copy-edit. |
| Muscle selection | Clarified that multiple muscles were harvested/scanned but soleus was selected for final whole-section analysis. | Acceptable; improves transparency. | Ensure this limitation is in Methods/Discussion. |
| Morphological filtering | Added area thresholds, circularity threshold ≥0.3, border-fiber exclusion. | Acceptable, though variable upper limits should be justified. | State rationale for variable upper thresholds. |
| Excluded fibers / flow diagram | Supplementary Table 1 and Fig. 1 reportedly provide QC counts and workflow. | Acceptable if the data are visible and easy to interpret. | Check Supplementary Table 1 is included with final submission. |
| Processing time | Reported average time and clarified that server latency varies. | Acceptable. | No further issue. |
| Data availability | Deposited raw per-fiber/per-animal CSA data, images, and masks in Zenodo. | Strong improvement. | Verify public access and file organization. |
| SAM citation | Full citation added with arXiv identifier. | Acceptable. | No further issue. |
| Exact p-values and violin plot summaries | Added exact p-values and defined median/quartiles in figure legends. | Acceptable, subject to statistical-unit clarification. | Ensure p-values align with valid inferential units. |

# 5. Remaining comments to authors

The revision is much improved. I recommend only minor revision focused on clarity and statistical framing. The following points should be addressed before acceptance:

**Clarify the unit of inference throughout the manuscript.** For each figure and statistical comparison, explicitly state whether n represents animals, images/sections, or fibers. Fiber-level plots may be retained as descriptive distributions, but biological conclusions should be based on animal-level or appropriately hierarchical inference.

**Refine the KS/distributional analyses.** If the KS tests are performed on pooled fiber observations, present them as exploratory/descriptive or justify them using an animal-level, bootstrapped, or hierarchical approach. Avoid language that suggests independent biological replication when the input observations are nested fibers.

**Report validation metrics more directly.** In addition to p-values comparing Dice coefficients, report the actual Dice values, preferably mean/median with SD/IQR and/or 95% confidence intervals. If IoU is not reported, state why Dice was chosen as the primary metric.

**Address the Bland-Altman/correlation request transparently.** The explanation that one-to-one fiber matching across modalities is difficult is reasonable. However, the manuscript should clearly acknowledge this as a limitation and, if feasible, provide image-level or animal-level CSA agreement metrics as a partial substitute.

**Moderate overclaims.** Replace phrases such as “decisively prove,” “human-level accuracy,” and “completely preventing subjective human bias” with more scientifically cautious phrasing such as “support,” “within the range of inter-observer variability,” and “reducing the risk of subjective bias.”

**Confirm archive completeness.** Ensure the Zenodo record contains raw per-fiber data, per-animal summary tables, representative input images, generated masks, the Fiji/ImageJ macro, local Python script, parameter documentation, README, and license/access statement.

**Strengthen limitations.** Add or refine limitations related to the small number of animals in the injury model, stain and scanner variability, possible performance differences in severe pathology/regenerating tissue, reliance on a hosted web service, and limited generalizability beyond mouse soleus sections.
