# Supplementary material for: Web-based Cellpose‑SAM segmentation enables quantification of cross-sectional area in formalin-fixed paraffin-embedded skeletal muscle sections stained with hematoxylin and eosin
Source: PLoS One. 2026 Sep 21;21(9):e0358476. doi: 10.1371/journal.pone.0358476 (PMC13592613; doi:10.1371/journal.pone.0358476)
Supplement: Supplementary file 7 [file pone.0358476.s007.docx]

**Authors’ Responses to Reviewer’s Comments**

The revision is much improved. I recommend only minor revision focused on clarity and statistical framing. The following points should be addressed before acceptance:

1. Clarify the unit of inference throughout the manuscript.

For each figure and statistical comparison, explicitly state whether n represents animals, images/sections, or fibers. Fiber-level plots may be retained as descriptive distributions, but biological conclusions should be based on animal-level or appropriately hierarchical inference.

(Response)

We thank the Reviewer for this helpful suggestion. We have revised every relevant figure legend and in-text statistic to explicitly specify what *n* represents in each comparison. Specifically, in Fig. 2, *n*=7 refers to whole cross-sectional images of the mid-belly region of the soleus muscle obtained from seven mice (Page 8, Lines 177-179; Page 14, Lines 301-303; Page 29, Lines 641-642). In Fig. 3B-E, Table 1, and Fig. S1, analyses were performed using 10 representative images selected from seven soleus muscle sections (1-2 representative images per section). The manual-to-manual (M-M) comparison was based on *n*=10 image pairs, whereas the CS-to-manual (CS-M) comparison was based on *n*=20 image pairs derived from these same 10 images. In Fig. 6E-H, *n*=3 mice (sham) and *n*=4 mice (SCI) indicate the number of animals analyzed, with one whole cross-sectional image of the mid-belly region of the soleus muscle obtained from each animal. In addition, the fiber-level plots (e.g., violin plots and relative frequency distributions in Figs. 3E, 4B, 4C, 5B, 5C, and 6H) are now described as descriptive visualizations of pooled fiber-level distributions rather than inferential statistical analyses. The corresponding descriptions have been revised consistently throughout texts and figure legends (Page 12, Lines 263-264; Page 16, Lines 352-356; Page 18, Line 411; Page 30, Lines 669-671, 682-683, and 685-686; Page 31, Lines 697-698 and 700-701; Page 32, 717-718). We believe these revisions now make the unit of inference explicit throughout the manuscript while clearly distinguishing descriptive fiber-level visualizations from inferential statistical analyses.

2. Refine the KS/distributional analyses.

If the KS tests are performed on pooled fiber observations, present them as exploratory/descriptive or justify them using an animal-level, bootstrapped, or hierarchical approach. Avoid language that suggests independent biological replication when the input observations are nested fibers.

(Response)

We agree with the Reviewer's comment. The KS analyses performed on pooled fiber-level CSA distributions (Figs. 3E, 4C, 5C, and 6H) have been explicitly reframed as descriptive comparisons rather than tests of independent biological replication. Accordingly, the corresponding texts and figure legends have been revised to clarify the descriptive nature of these pooled fiber-level distributions (Page 12, Lines 263-264; Page 16, Lines 352-356; Page 18, Line 411; Page 30, Lines 669-671 and 685-686; Page 31, 700-701; Page 32, Lines 717-718). We have also revised the manuscript to avoid language implying that pooled fiber-level observations represent independent biological replication, and all biological conclusions are now based on animal- or image-level analyses, as appropriate.

3. Report validation metrics more directly.

In addition to p-values comparing Dice coefficients, report the actual Dice values, preferably mean/median with SD/IQR and/or 95% confidence intervals. If IoU is not reported, state why Dice was chosen as the primary metric.

(Response)

We thank the Reviewer for this helpful suggestion. We have added a new Table 1 reporting the actual Dice coefficient and IoU values as the median and interquartile range (Q1-Q3) for both the manual-to-manual (M-M) and CS-to-manual (CS-M) comparisons in H&E- and WGA-stained sections (Page 33). In addition, the corresponding IoU results have been incorporated into the Results section (Page 15, Lines 335-339; Page 16, Line 350) and are presented in the new Fig. S1.

4. Address the Bland-Altman/correlation request transparently.

The explanation that one-to-one fiber matching across modalities is difficult is reasonable. However, the manuscript should clearly acknowledge this as a limitation and, if feasible, provide image-level or animal-level CSA agreement metrics as a partial substitute.

(Response)

In response to the Reviewer's comment, we have added a statement to a newly included Limitations paragraph in the Discussion (Pages 21-22, Lines 478-501) clarifying that our cross-stain and cross-modality comparisons (H&E vs. TB and H&E vs. WGA) were performed using image- or animal-level summary measurements rather than direct one-to-one correspondence of individual myofibers. We further clarified that accurate fiber-level matching across separately stained serial sections remains challenging because different staining protocols generally require adjacent serial sections rather than the same tissue section, and that Bland-Altman analysis was therefore not performed. As a partial substitute, we now specify that image-level mean CSA comparisons and animal-level mean and median CSA measurements were used as complementary summary-level assessments of morphometric consistency and biological differences, respectively.

5. Moderate overclaims.

Replace phrases such as “decisively prove,” “human-level accuracy,” and “completely preventing subjective human bias” with more scientifically cautious phrasing such as “support,” “within the range of inter-observer variability,” and “reducing the risk of subjective bias.”

(Response)

We have revised the manuscript language accordingly throughout the Results and Discussion sections. Specifically, "achieved human-level accuracy" has been changed to "within the range of inter-observer variability" (Page 15, Lines 337-338); "ensuring highly accurate and unbiased quantification" has been changed to "reducing the risk of subjective bias" (Page 10, Line 228); "highly effective substitute" has been changed to "practical substitute" (Page 20, Line 449); "equivalent between H&E- and TB-stained sections" has been changed to "comparable between H&E- and TB-stained sections" (Page 20, Lines 450-451). We believe these revisions provide a more balanced interpretation of our findings and align the language more closely with the strength of the supporting evidence.

6. Confirm archive completeness.

Ensure the Zenodo record contains raw per-fiber data, per-animal summary tables, representative input images, generated masks, the Fiji/ImageJ macro, local Python script, parameter documentation, README, and license/access statement.

(Response)

We have revised the Zenodo archive (https://doi.org/10.5281/zenodo.21482173), which is publicly accessible. The archive now contains a single Excel file (*ParkJH_PO_raw data.xlsx*) that includes the raw data underlying all quantitative analyses presented in Figs. 2-6 and Fig. S1, including raw per-fiber CSA measurements, image-level measurements, and per-animal summary data. It also contains representative input images, generated segmentation masks, the custom Fiji/ImageJ macro, the local Python script, parameter documentation, and a README file. We have also added the direct Zenodo URL alongside the DOI in the Code and Data Availability section to facilitate reviewer and reader access.

7. Strengthen limitations.

Add or refine limitations related to the small number of animals in the injury model, stain and scanner variability, possible performance differences in severe pathology/regenerating tissue, reliance on a hosted web service, and limited generalizability beyond mouse soleus sections.

(Response)

In response to the Reviewer’s comment, we have added a dedicated Limitations paragraph at the Discussion (Pages 21-22, Lines 478-501). Specifically, we discussed the limited number of animals evaluated in the denervation injury model (sham, *n*=3; SCI, *n*=4), the use of a single slide scanner, staining protocol, and mouse strain for validation, the lack of specific evaluation in severely dystrophic, necrotic, or actively regenerating muscle, the limitation of image- and animal-level rather than direct fiber-level cross-stain and cross-modality validation, and the current evaluation being limited to mouse soleus muscle. In addition, we acknowledge that the primary workflow relies on a publicly hosted web interface, although a local Python implementation is also provided as an alternative. These revisions provide a more balanced discussion of the limitations and potential applicability of the proposed workflow.
