# Supplementary material for: Web-based Cellpose‑SAM segmentation enables quantification of cross-sectional area in formalin-fixed paraffin-embedded skeletal muscle sections stained with hematoxylin and eosin
Source: PLoS One. 2026 Sep 21;21(9):e0358476. doi: 10.1371/journal.pone.0358476 (PMC13592613; doi:10.1371/journal.pone.0358476)
Supplement: Peer review history

# Peer review: Decision Letter 0

**DOI:** 10.1371/journal.pone.0358476.r001

**Authors:** Masoud Rahmati

25 Feb 2026

Dear Dr. KIM,

Thank you for submitting your manuscript to PLOS ONE. After careful consideration, we feel that it has merit but does not fully meet PLOS ONE’s publication criteria as it currently stands. Therefore, we invite you to submit a revised version of the manuscript that addresses the points raised during the review process.

==============================

Please submit your revised manuscript by Apr 11 2026 11:59PM. If you will need more time than this to complete your revisions, please reply to this message or contact the journal office at plosone@plos.org. When you're ready to submit your revision, log on to https://www.editorialmanager.com/pone/ and select the 'Submissions Needing Revision' folder to locate your manuscript file.

- A letter that responds to each point raised by the academic editor and reviewer(s). You should upload this letter as a separate file labeled 'Response to Reviewers'.
- A marked-up copy of your manuscript that highlights changes made to the original version. You should upload this as a separate file labeled 'Revised Manuscript with Track Changes'.
- An unmarked version of your revised paper without tracked changes. You should upload this as a separate file labeled 'Manuscript'.

If you would like to make changes to your financial disclosure, please include your updated statement in your cover letter. Guidelines for resubmitting your figure files are available below the reviewer comments at the end of this letter.

If applicable, we recommend that you deposit your laboratory protocols in protocols.io to enhance the reproducibility of your results. Protocols.io assigns your protocol its own identifier (DOI) so that it can be cited independently in the future. For instructions see: https://journals.plos.org/plosone/s/submission-guidelines#loc-laboratory-protocols. Additionally, PLOS ONE offers an option for publishing peer-reviewed Lab Protocol articles, which describe protocols hosted on protocols.io. Read more information on sharing protocols at https://plos.org/protocols?utm_medium=editorial-email&utm_source=authorletters&utm_campaign=protocols.

We look forward to receiving your revised manuscript.

Kind regards,

Masoud Rahmati

Academic Editor

PLOS One

Journal Requirements:

When submitting your revision, we need you to address these additional requirements.

1. Please ensure that your manuscript meets PLOS ONE's style requirements, including those for file naming. The PLOS ONE style templates can be found at

https://journals.plos.org/plosone/s/file?id=wjVg/PLOSOne_formatting_sample_main_body.pdf and

https://journals.plos.org/plosone/s/file?id=ba62/PLOSOne_formatting_sample_title_authors_affiliations.pdf

2. Please note that PLOS One has specific guidelines on code sharing for submissions in which author-generated code underpins the findings in the manuscript. In these cases, we expect all author-generated code to be made available without restrictions upon publication of the work. Please review our guidelines at https://journals.plos.org/plosone/s/materials-and-software-sharing#loc-sharing-code and ensure that your code is shared in a way that follows best practice and facilitates reproducibility and reuse.

3. Thank you for stating the following financial disclosure:

“This work was supported by the 2023 Yeungnam University Research Grant (to J.-R.K.).”

Please state what role the funders took in the study.  If the funders had no role, please state: "The funders had no role in study design, data collection and analysis, decision to publish, or preparation of the manuscript."

If this statement is not correct you must amend it as needed.

Please include this amended Role of Funder statement in your cover letter; we will change the online submission form on your behalf

4.PLOS requires an ORCID iD for the corresponding author in Editorial Manager on papers submitted after December 6th, 2016. Please ensure that you have an ORCID iD and that it is validated in Editorial Manager. To do this, go to ‘Update my Information’ (in the upper left-hand corner of the main menu), and click on the Fetch/Validate link next to the ORCID field. This will take you to the ORCID site and allow you to create a new iD or authenticate a pre-existing iD in Editorial Manager.

5. Please include captions for your Supporting Information files at the end of your manuscript, and update any in-text citations to match accordingly. Please see our Supporting Information guidelines for more information: http://journals.plos.org/plosone/s/supporting-information.

6. If the reviewer comments include a recommendation to cite specific previously published works, please review and evaluate these publications to determine whether they are relevant and should be cited. There is no requirement to cite these works unless the editor has indicated otherwise.

[Note: HTML markup is below. Please do not edit.]

Reviewers' comments:

Reviewer's Responses to Questions

**Comments to the Author**

1. Is the manuscript technically sound, and do the data support the conclusions?

Reviewer #1: Partly

**********

2. Has the statistical analysis been performed appropriately and rigorously?

Reviewer #1: No

**********

3. Have the authors made all data underlying the findings in their manuscript fully available?

The PLOS Data policy

Reviewer #1: No

**********

4. Is the manuscript presented in an intelligible fashion and written in standard English?

Reviewer #1: Yes

**********

Reviewer #1: Dear Authors,

Thank you for submitting your manuscript. Overall, the study presents a feasible and generally well-described workflow for myofiber segmentation and morphometric quantification on routine bright-field stains (e.g., H&E/TB) using a web-based segmentation step followed by Fiji/ImageJ analysis. The biological direction of your findings in the denervation model (reduced CSA and a left-shift in distributions compared with sham) is plausible and supports a limited conclusion that the workflow can detect expected atrophy-related changes.

That said, several issues currently prevent the manuscript from fully supporting stronger claims (e.g., “accuracy,” “robustness,” or broad reproducibility). First, the work would benefit from quantitative validation against a ground truth-for example, expert manual annotations and/or a commonly used reference method such as laminin/collagen IV immunostaining on cryosections. Including objective segmentation performance metrics (e.g., Dice/IoU, boundary error, and agreement in CSA via Bland-Altman or equivalent) would substantially strengthen the technical evidence.

Second, the statistical approach requires refinement to avoid pseudoreplication. Fibers are nested within sections and animals, yet the inferential testing appears to treat fibers as independent observations. This can artificially inflate precision and produce overly small p-values, particularly with modest animal numbers. Please consider reanalyzing the primary outcomes using the animal as the unit of inference (e.g., per-animal summary measures) and/or applying mixed-effects models that account for clustering. If H&E and TB data are derived from the same animals/adjacent sections, a paired analysis at the appropriate level should be considered. Reporting effect sizes with confidence intervals at the animal/section level would improve interpretability.

Third, the manuscript does not yet make the full underlying data readily available to enable reproducibility. To align with open science expectations, please provide (or deposit in a stable repository) the per-fiber measurement tables linked to animal/section, key analysis scripts/macros, and-where feasible-representative image tiles and corresponding segmentation masks/ROIs (or clearly justify any restrictions).

Finally, the manuscript is generally intelligible and written in standard English. To improve clarity and reproducibility further, I recommend explicitly detailing: the number of animals, sections per animal, the image selection strategy (whole-section vs sampled fields), and the exact QC/curation steps and parameters used in the segmentation and downstream quantification.

Addressing the validation, statistical hierarchy, and data availability points above would greatly strengthen the manuscript and allow your conclusions to be stated with higher confidence.

**********

what does this mean?). If published, this will include your full peer review and any attached files.

If you choose “no”, your identity will remain anonymous but your review may still be made public.

**Do you want your identity to be public for this peer review?**  For information about this choice, including consent withdrawal, please see our Privacy Policy

Reviewer #1: No

**********

[NOTE: If reviewer comments were submitted as an attachment file, they will be attached to this email and accessible via the submission site. Please log into your account, locate the manuscript record, and check for the action link "View Attachments". If this link does not appear, there are no attachment files.]

To ensure your figures meet our technical requirements, please review our figure guidelines: https://journals.plos.org/plosone/s/figures

You may also use PLOS’s free figure tool, NAAS, to help you prepare publication quality figures: https://journals.plos.org/plosone/s/figures#loc-tools-for-figure-preparation.

NAAS will assess whether your figures meet our technical requirements by comparing each figure against our figure specifications.

Attachment Submitted filename: Peer Review Report PONE-D-25-67815.docx

# Author comment: Author response to Decision Letter 1

**DOI:** 10.1371/journal.pone.0358476.r002

9 Jun 2026

Authors’ Responses to Reviewers’ Comments

Major comments

1. CSA units are inconsistent between text and figures.

Results text reports CSA in µm² (e.g., ~1288 µm²), while Figures 2B and 3B label the CSA axis as 'CSA (mm²)' while adjacent distribution panels use µm². Given the plotted ranges (0–5000 or 0–10000), the correct unit is almost certainly µm², not mm².

Please correct all axis labels, legends, and any related text to ensure consistent units throughout; re-check any derived interpretations.

(Response)

We sincerely thank the reviewer for catching this typographical error. The reviewer is absolutely correct; the proper unit for myofiber CSA is µm², not mm². We have carefully corrected the y-axis labels in Figs. 4B and 5B in the revised manuscript. Furthermore, as requested, we have double-checked all derived data and interpretations. We confirm that this was strictly a visual labeling error; all underlying CSA measurements, statistical outcomes, and interpretations were already correctly based on µm² and remain entirely unaffected.

2. Statistical unit of analysis and pseudo-replication

Statistical Analysis states unpaired t-tests and KS tests; Results use large fiber counts (hundreds) as n.

Issue: Fibers are nested within animals/sections, so treating each fiber as an independent observation inflates the effective sample size and can produce spuriously small p-values.

Reanalyze using animal-level inference (e.g., per-animal mean/median CSA and compare groups with n=number of mice) and/or mixed-effects models with random intercepts for animal (and section/tile if relevant). For distributional comparisons, use animal-level bootstrapping or hierarchical methods. Report effect sizes with 95% confidence intervals and clearly define the biological replicate.

(Response)

We thank the reviewer for highlighting the issue of pseudo-replication. We agree that individual fibers are nested within sections/animals and therefore are not independent biological replicates; treating fibers as independent observations can inflate the effective sample size and yield spuriously small p-values.

Accordingly, we reanalyzed CSA using animal-level inference, defining the biological replicate as the individual mouse (Sham, n=3; SCI, n=4). Given the small number of animals, we focused on conservative animal-level inference (per-animal summaries) as the primary analysis to directly reflect the biological replication structure. For each animal, we pooled all segmented fibers to compute a single per-animal mean and per-animal median CSA, and performed group comparisons using n=number of animals. The updated figures show fiber distributions for each animal (Fig. 6E) and the per-animal summary (mean or median) values used for inference (Fig. 6F and 6G).

Using this animal-level approach, the SCI group still exhibited a significant reduction compared to the sham group (Fig. 6E-6H). We now report effect sizes with 95% confidence intervals (CIs) based on these animal-level summaries. The revised Results section can be found on Pages 17-18, Lines 393–397.

Finally, the relative frequency distributions derived from multiple samples were re-evaluated using the Kolmogorov-Smirnov test, and the exact p-value was included in Fig. 6H.

3. Add quantitative validation of segmentation accuracy/agreement.

Feasibility and stain concordance are shown, but accuracy is not quantified against a reference standard.

On a representative subset of images (multiple animals, stains, tissue types), compare Cellpose-SAM outputs to (i) manual ground-truth annotations and/or (ii) immunofluorescence-based basal lamina segmentation. Report Dice/IoU (or similar), split/merge error rates, and downstream CSA agreement (Bland–Altman bias/limits, correlation). Also report how many fibers required manual correction/exclusion.

(Response)

We sincerely thank the reviewer for pointing out the critical need for quantitative validation against a reference standard. We fully agree with this assessment. To address this, we have performed the requested quantitative analyses and updated the manuscript as detailed below.

i) Comparisons Cellpose-SAM outputs to manual ground-truth annotations and/or immunofluorescence-based basal lamina segmentation. Report Dice/IoU and assessment of split/merge errors.

1) Assessment of split/merge errors via Dice coefficient

To functionally address split or merge errors, we utilized the Dice coefficient, which robustly captures these spatial deviations. Rather than reporting an isolated error rate, we directly compared the accuracy of Cellpose-SAM (CS) outputs (CS-to-Manual, CS-M) against inherent human variability (Manual-to-Manual, M-M baseline).

2) Comparison to manual ground-truth (H&E analysis)

First, we validated the automated Cellpose-SAM (CS) outputs on routine H&E-stained images by comparing them against manual (M) tracings. To establish a rigorous reference standard, manual annotations for representative images (n=10) were independently performed by two investigators (M1 and M2) to define the M-M baseline. When comparing the automated CS outputs to these annotations, there was no significant difference in the Dice coefficient between the M-M baseline and the CS-M group (p=0.1971) in the revised Figs. 3A and 3B. This concisely confirms that any minor deviations generated by Cellpose-SAM (such as split/merge tendencies) strictly fall within the normal range of human observer variability, achieving human-level accuracy.

3) Comparison to ECM/basal lamina segmentation (WGA analysis)

Second, addressing the reviewer’s point regarding immunofluorescence-based basal lamina segmentation, traditional markers (e.g., laminin or collagen IV) are often restricted to cryosections due to epitope masking during FFPE procedures. Therefore, to establish a robust reference standard for true myofiber boundaries in FFPE tissues, we utilized WGA staining, a lectin that reliably binds to sarcolemmal glycoproteins and the extracellular matrix (PMID: 21181705). Applying the exact same validation design (M-M vs. CS-M) to WGA-stained images, the automated CS segmentation similarly showed no significant difference compared to manual tracing (p=0.1179; Fig. 3C). Furthermore, the choice of staining (H&E vs. WGA) did not significantly affect the mean cross-sectional area (CSA) or their relative frequency distributions (Fig. 3D and 3E).

iii) Report downstream CSA agreement (Bland–Altman bias/limits, correlation)

Initially, we tried to perform a fiber-to-fiber correlation and Bland-Altman analysis as suggested. However, directly matching hundreds of individual fibers 1:1 across different modalities (e.g., consecutive sections of H&E and WGA) introduces unavoidable spatial registration artifacts. Therefore, we utilized an alternative, highly comprehensive approach to robustly validate the downstream morphometric agreement:

• Mean CSA: The automated pipeline showed no significant difference in the mean CSA between H&E and WGA images (p=0.0848, Fig. 3D).

• Fiber size distribution: More importantly, we evaluated the fiber size frequency distribution across the entire pooled dataset of individual fibers. This method is highly sensitive to segmentation biases; for instance, split errors would unnaturally skew the curve to the left, while merge errors would skew it to the right. As depicted in Fig. 3E, the frequency curves of H&E overlapped with the those of WGA. Statistical analysis confirmed no significant difference between the two distribution patterns (p=0.9994).

We have fully detailed these validation procedures in the revised Results section (Pages 14-15, Lines 315-346) and Fig. 3.

iv) Report how many fibers required manual correction/exclusion:

To provide full transparency regarding the post-processing steps as requested, we have quantified the exact number of fibers requiring manual correction or exclusion after the initial segmentation by Cellpose-SAM and subsequent automated filtering via our custom macro. These manual intervention metrics, evaluated on the 10 H&E and WGA-stained images used for manual ground-truth annotations in Fig. 3, are detailed in the Table. Overall, the Table highlights that manual intervention is rarely needed after our automated segmentation. With manual modification rates remaining exceptionally low (0-1.4% for H&E and 0-5.3% for WGA), these metrics decisively prove the high precision and reliability of our Cellpose-SAM and macro-driven workflow.

Table. Evaluation of manual interventions required post-Cellpose-SAM and macro filtering.

Staining Image number Original ROIs Manual modification ROIs Final ROIs Modification rate (%)

H&E 1 47 0 47 0

2 57 0 57 0

3 72 0 72 0

4 64 0 64 0

5 65 0 65 0

6 65 0 65 0

7 70 1 69 1.4

8 70 1 69 1.4

9 62 0 62 0

10 61 0 61 0

WGA 1 46 0 46 0

2 54 0 54 0

3 69 0 69 0

4 62 2 60 3.2

5 70 2 68 2.7

6 70 0 70 0

7 72 4 68 5.3

8 69 0 69 0

9 61 1 60 1.6

10 61 1 60 1.6

In addition, we presented manual correction rate in whole soleus muscle cross-sections obtained from mice (Sham, n=3 and SCI, n=4). As detailed in the newly added Supplementary Table 1, the need for manual intervention was minimal. Across all analyzed whole-muscle images, manually added fibers ranged from only 0 to 42, and manually excluded fibers ranged from 2 to 12 per image. Notably, the overall manual correction rate remained exceedingly low, ranging from 0.54% to 4.01%. These quantitative results strongly demonstrate the high accuracy and out-of-the-box robustness of our web-based Cellpose-SAM segmentation pipeline prior to any human intervention.

In conclusion, these comprehensive validations confirm that our web-based Cellpose-SAM pipeline captures the true morphometric profile without introducing systematic sizing biases. Ultimately, it establishes that routine H&E staining, driven by our web-based Cellpose-SAM workflow, serves as a highly effective and accessible substitute for traditional fluorescence-based segmentation.

4. Reproducibility risks from reliance on a hosted web app; improve parameter reporting and archiving.

Methods indicate use of a Hugging Face Cellpose-SAM interface and 'default settings'.

Issue: Hosted services and defaults can change over time; 'default' is not reproducible unless fully specified. Provide complete parameter values (as shown in the interface), exact model identifiers, and any version/commit information available. In addition to GitHub, create an archived release (e.g., Zenodo DOI) of the macro and documentation. Consider providing a local/scriptable alternative (Python command line or container) for long-term reproducibility.

(Response)

We sincerely thank the reviewer for raising this critical point regarding long-term reproducibility and open science. We fully agree that relying solely on a third-party hosted web interface and stating "default settings" is insufficient, as web applications and defaults are subject to change over time. To ensure robust and permanent reproducibility, we have addressed all reviewer’s recommendations and updated the Methods section accordingly:

i) Complete parameter values and exact model identifiers

We have revised the Methods section to explicitly detail the exact parameters and model identifiers used in the version 1.2.0 interface. Rather than older segmentation models, the web application utilizes the unified Cellpose-SAM model (identifier: cpsam), which is built upon the ViT-L (Vision Transformer-Large) backbone architecture from the Segment Anything Model. The default segmentation parameters were applied strictly without any modifications, and they are now fully specified in the manuscript as follows: max resize = 1000, max iter = 250, flow thresh = 0.4, and cellprob thresh = 0. These details are stated on Page 9, Lines 197-202, and in Fig. 1B.

ii) Local/scriptable alternative for long-term reproducibility

We completely agree with the potential risks of hosted services. To guarantee long-term reproducibility regardless of the Hugging Face interface's status, we have developed a local, scriptable alternative Python script (run_cellpose_local.py). This allows users to easily execute the exact Cellpose-SAM pipeline locally on their own machines using the identical parameters reported in our study.

iii) Archival release via Zenodo

To ensure the immutability and permanent availability of our complete workflow, we have created an archived release containing the local Python script, our custom Fiji/ImageJ macro, detailed user documentation, representative images, and raw data files. This repository is now permanently archived and publicly accessible via Zenodo (DOI: 10.5281/zenodo.19447533).

5. Whole-slide image handling is unclear given file-size limits; specify tiling/downsampling and aggregation.

Methods note a 10 MB upload limit. Whole-slide 40× images usually exceed this, yet Results imply whole-section analysis. Describe the exact export strategy (downsampling factor, tile size/overlap, number of tiles per section, how boundary fibers were handled to avoid double counting). If downsampled images were used, quantify the impact on CSA bias and provide recommended minimal resolution.

(Response)

We deeply appreciate the reviewer’s careful attention to whole-slide image (WSI) handling and the potential for resolution-dependent CSA bias. We would like to clarify our exact export strategy: while we did apply a moderate downsampling factor, our analysis did not involve any image splitting or tiling.

i) Export strategy (Single image with a downsampling factor of 4)

Unlike massive human tissue sections, the entire cross-section of a mouse soleus muscle is anatomically small. To export the whole section as a single, contiguous image while strictly adhering to the platform's file-size limits, we applied a downsampling factor of 4 during export. Because the entire section was processed as a single image, there were no grid tiles to merge, and consequently, absolutely no risk of double-counting fibers. Furthermore, any incomplete boundary fibers touching the absolute extreme edges of the single whole-section image were automatically excluded using the 'Remove Border Labels' function in MorphoLibJ.

ii) Optimization of downsampling and CSA bias

To address the reviewer's valid concern regarding downsampling-induced CSA bias, we have added our systematic multi-resolution optimization data in Fig. 2. We systematically exported WSI resolutions from D1 (4.00 pixel/μm) down to D16 (0.25 pixel/μm) via QuPath and analyzed them. We purposefully selected D4 (1.00 pixel/μm) as the operational reference. This specific downsampling factor effectively suppressed intracellular textural noise (which impairs deep-learning segmentation) while retaining robust boundary fidelity. Crucially, our quantitative validation confirmed that the D4 resolution (yielding final WSI resolutions generally ranging from 0.8 to 1.0 pixel/μm) introduced no significant morphometric bias in CSA calculations compared to the native resolution. To ensure absolute transparency, the exact spatial resolution for each analyzed image has been explicitly detailed in the figure legends of Fig. 2A. To answer reviewer’s final point, we recommend a minimal spatial resolution of 0.8 pixel/μm (ideal range: 0.8-2.0 pixel/μm) for the web-based Cellpose-SAM workflow.

These details are stated on Pages 13-14, Lines 292-313, and in Fig. 2.

6. Pixel calibration and measurement integrity must be explicitly documented.

CSA computation requires correct pixel-to-micron calibration in Fiji/ImageJ.

Specify how scale calibration was set (scanner metadata, known calibration, scale-bar reference) and ensure the macro enforces calibration consistently across all images. Include a simple calibration QC step and report tolerance.

(Response)

We deeply appreciate the reviewer’s highly constructive feedback regarding pixel calibration. We completely agree that these technical parameters are crucial for measurement integrity. We have extensively revised the Methods section (under the new subheadings “Image preparation and downsampling optimization” on Pages 8-9, Lines 166-192 and “Pixel (px) calibration, morphological filtering, and CSA measurement” on Page 10, Line

Attachment Submitted filename: Authors responses to reviewers comments.docx

# Peer review: Decision Letter 1

**DOI:** 10.1371/journal.pone.0358476.r003

**Authors:** Masoud Rahmati

7 Jul 2026

Dear Dr. KIM,

Thank you for submitting your manuscript to PLOS ONE. After careful consideration, we feel that it has merit but does not fully meet PLOS ONE’s publication criteria as it currently stands. Therefore, we invite you to submit a revised version of the manuscript that addresses the points raised during the review process.

==============================

Please submit your revised manuscript by Aug 21 2026 11:59PM. If you will need more time than this to complete your revisions, please reply to this message or contact the journal office at plosone@plos.org. When you're ready to submit your revision, log on to https://www.editorialmanager.com/pone/ and select the 'Submissions Needing Revision' folder to locate your manuscript file.

- A letter that responds to each point raised by the academic editor and reviewer(s). You should upload this letter as a separate file labeled 'Response to Reviewers'.
- A marked-up copy of your manuscript that highlights changes made to the original version. You should upload this as a separate file labeled 'Revised Manuscript with Track Changes'.
- An unmarked version of your revised paper without tracked changes. You should upload this as a separate file labeled 'Manuscript'.

If you would like to make changes to your financial disclosure, please include your updated statement in your cover letter. Guidelines for resubmitting your figure files are available below the reviewer comments at the end of this letter.

If applicable, we recommend that you deposit your laboratory protocols in protocols.io to enhance the reproducibility of your results. Protocols.io assigns your protocol its own identifier (DOI) so that it can be cited independently in the future. For instructions see: https://journals.plos.org/plosone/s/submission-guidelines#loc-laboratory-protocols. Additionally, PLOS ONE offers an option for publishing peer-reviewed Lab Protocol articles, which describe protocols hosted on protocols.io. Read more information on sharing protocols at https://plos.org/protocols?utm_medium=editorial-email&utm_source=authorletters&utm_campaign=protocols.

As the corresponding author, your ORCID iD is verified in the submission system and will appear in the published article. PLOS supports the use of ORCID, and we encourage all coauthors to register for an ORCID iD and use it as well. Please encourage your coauthors to verify their ORCID iD within the submission system before final acceptance, as unverified ORCID iDs will not appear in the published article. *Only*  the individual author can complete the verification step; PLOS staff *cannot*  verify ORCID iDs on behalf of authors.

We look forward to receiving your revised manuscript.

Kind regards,

Masoud Rahmati

Academic Editor

PLOS One

Journal Requirements:

If the reviewer comments include a recommendation to cite specific previously published works, please review and evaluate these publications to determine whether they are relevant and should be cited. There is no requirement to cite these works unless the editor has indicated otherwise.

Please review your reference list to ensure that it is complete and correct. If you have cited papers that have been retracted, please include the rationale for doing so in the manuscript text, or remove these references and replace them with relevant current references. Any changes to the reference list should be mentioned in the rebuttal letter that accompanies your revised manuscript. If you need to cite a retracted article, indicate the article’s retracted status in the References list and also include a citation and full reference for the retraction notice.

[Note: HTML markup is below. Please do not edit.]

Reviewers' comments:

Reviewer's Responses to Questions

**Comments to the Author**

Reviewer #1: (No Response)

**********

2. Is the manuscript technically sound, and do the data support the conclusions?

Reviewer #1: Yes

**********

3. Has the statistical analysis been performed appropriately and rigorously?

Reviewer #1: Yes

**********

4. Have the authors made all data underlying the findings in their manuscript fully available?

The PLOS Data policy

Reviewer #1: Yes

**********

5. Is the manuscript presented in an intelligible fashion and written in standard English?

Reviewer #1: Yes

**********

Reviewer #1: The revised manuscript is substantially improved compared with the version that received major revision. The authors have corrected the obvious unit error, reanalyzed the key injury comparison using animal-level inference, added quantitative segmentation validation, strengthened reproducibility through parameter reporting and an archived release, clarified whole-slide export/downsampling, documented calibration, and resolved the laterality/blinding ambiguity in the sciatic nerve crush model.

From a research process perspective, the revision demonstrates good faith engagement with the reviewer comments and materially strengthens the credibility, reproducibility, and interpretability of the study.

The main remaining concern is not the feasibility of the method but the precision with which the authors frame inference. Fiber-level distributions are valuable descriptively, but biological inference should remain anchored at the animal or image/section level. The revised manuscript should ensure that all figures, legends, and statistical statements clearly distinguish descriptive fiber-level distributions from inferential comparisons based on biological replicates.

*Remaining comments to authors*

The following points should be addressed before acceptance:

Clarify the unit of inference throughout the manuscript. For each figure and statistical comparison, explicitly state whether n represents animals, images/sections, or fibers. Fiber-level plots may be retained as descriptive distributions, but biological conclusions should be based on animal-level or appropriately hierarchical inference.

Refine the KS/distributional analyses. If the KS tests are performed on pooled fiber observations, present them as exploratory/descriptive or justify them using an animal-level, bootstrapped, or hierarchical approach. Avoid language that suggests independent biological replication when the input observations are nested fibers.

Report validation metrics more directly. In addition to p-values comparing Dice coefficients, report the actual Dice values, preferably mean/median with SD/IQR and/or 95% confidence intervals. If IoU is not reported, state why Dice was chosen as the primary metric.

Address the Bland-Altman/correlation request transparently. The explanation that one-to-one fiber matching across modalities is difficult is reasonable. However, the manuscript should clearly acknowledge this as a limitation and, if feasible, provide image-level or animal-level CSA agreement metrics as a partial substitute.

Moderate overclaims. Replace phrases such as “decisively prove,” “human-level accuracy,” and “completely preventing subjective human bias” with more scientifically cautious phrasing such as “support,” “within the range of inter-observer variability,” and “reducing the risk of subjective bias.”

Confirm archive completeness. Ensure the Zenodo record contains raw per-fiber data, per-animal summary tables, representative input images, generated masks, the Fiji/ImageJ macro, local Python script, parameter documentation, README, and license/access statement.

Strengthen limitations. Add or refine limitations related to the small number of animals in the injury model, stain and scanner variability, possible performance differences in severe pathology/regenerating tissue, reliance on a hosted web service, and limited generalizability beyond mouse soleus sections.

**********

what does this mean?). If published, this will include your full peer review and any attached files.

If you choose “no”, your identity will remain anonymous but your review may still be made public.

**Do you want your identity to be public for this peer review?**  For information about this choice, including consent withdrawal, please see our Privacy Policy

Reviewer #1: No

**********

[NOTE: If reviewer comments were submitted as an attachment file, they will be attached to this email and accessible via the submission site. Please log into your account, locate the manuscript record, and check for the action link "View Attachments". If this link does not appear, there are no attachment files.]

To ensure your figures meet our technical requirements, please review our figure guidelines: https://journals.plos.org/plosone/s/figures

You may also use PLOS’s free figure tool, NAAS, to help you prepare publication quality figures: https://journals.plos.org/plosone/s/figures#loc-tools-for-figure-preparation.

NAAS will assess whether your figures meet our technical requirements by comparing each figure against our figure specifications.

Attachment Submitted filename: Re-review report OK.docx

# Author comment: Author response to Decision Letter 2

**DOI:** 10.1371/journal.pone.0358476.r004

10 Aug 2026

Authors’ Responses to Reviewer’s Comments

The revision is much improved. I recommend only minor revision focused on clarity and statistical framing. The following points should be addressed before acceptance:

1. Clarify the unit of inference throughout the manuscript.

For each figure and statistical comparison, explicitly state whether n represents animals, images/sections, or fibers. Fiber-level plots may be retained as descriptive distributions, but biological conclusions should be based on animal-level or appropriately hierarchical inference.

(Response)

We thank the Reviewer for this helpful suggestion. We have revised every relevant figure legend and in-text statistic to explicitly specify what n represents in each comparison. Specifically, in Fig. 2, n=7 refers to whole cross-sectional images of the mid-belly region of the soleus muscle obtained from seven mice (Page 8, Lines 177-179; Page 14, Lines 301-303; Page 29, Lines 641-642). In Fig. 3B-E, Table 1, and Fig. S1, analyses were performed using 10 representative images selected from seven soleus muscle sections (1-2 representative images per section). The manual-to-manual (M-M) comparison was based on n=10 image pairs, whereas the CS-to-manual (CS-M) comparison was based on n=20 image pairs derived from these same 10 images. In Fig. 6E-H, n=3 mice (sham) and n=4 mice (SCI) indicate the number of animals analyzed, with one whole cross-sectional image of the mid-belly region of the soleus muscle obtained from each animal. In addition, the fiber-level plots (e.g., violin plots and relative frequency distributions in Figs. 3E, 4B, 4C, 5B, 5C, and 6H) are now described as descriptive visualizations of pooled fiber-level distributions rather than inferential statistical analyses. The corresponding descriptions have been revised consistently throughout texts and figure legends (Page 12, Lines 263-264; Page 16, Lines 352-356; Page 18, Line 411; Page 30, Lines 669-671, 682-683, and 685-686; Page 31, Lines 697-698 and 700-701; Page 32, 717-718). We believe these revisions now make the unit of inference explicit throughout the manuscript while clearly distinguishing descriptive fiber-level visualizations from inferential statistical analyses.

2. Refine the KS/distributional analyses.

If the KS tests are performed on pooled fiber observations, present them as exploratory/descriptive or justify them using an animal-level, bootstrapped, or hierarchical approach. Avoid language that suggests independent biological replication when the input observations are nested fibers.

(Response)

We agree with the Reviewer's comment. The KS analyses performed on pooled fiber-level CSA distributions (Figs. 3E, 4C, 5C, and 6H) have been explicitly reframed as descriptive comparisons rather than tests of independent biological replication. Accordingly, the corresponding texts and figure legends have been revised to clarify the descriptive nature of these pooled fiber-level distributions (Page 12, Lines 263-264; Page 16, Lines 352-356; Page 18, Line 411; Page 30, Lines 669-671 and 685-686; Page 31, 700-701; Page 32, Lines 717-718). We have also revised the manuscript to avoid language implying that pooled fiber-level observations represent independent biological replication, and all biological conclusions are now based on animal- or image-level analyses, as appropriate.

3. Report validation metrics more directly.

In addition to p-values comparing Dice coefficients, report the actual Dice values, preferably mean/median with SD/IQR and/or 95% confidence intervals. If IoU is not reported, state why Dice was chosen as the primary metric.

(Response)

We thank the Reviewer for this helpful suggestion. We have added a new Table 1 reporting the actual Dice coefficient and IoU values as the median and interquartile range (Q1-Q3) for both the manual-to-manual (M-M) and CS-to-manual (CS-M) comparisons in H&E- and WGA-stained sections (Page 33). In addition, the corresponding IoU results have been incorporated into the Results section (Page 15, Lines 335-339; Page 16, Line 350) and are presented in the new Fig. S1.

4. Address the Bland-Altman/correlation request transparently.

The explanation that one-to-one fiber matching across modalities is difficult is reasonable. However, the manuscript should clearly acknowledge this as a limitation and, if feasible, provide image-level or animal-level CSA agreement metrics as a partial substitute.

(Response)

In response to the Reviewer's comment, we have added a statement to a newly included Limitations paragraph in the Discussion (Pages 21-22, Lines 478-501) clarifying that our cross-stain and cross-modality comparisons (H&E vs. TB and H&E vs. WGA) were performed using image- or animal-level summary measurements rather than direct one-to-one correspondence of individual myofibers. We further clarified that accurate fiber-level matching across separately stained serial sections remains challenging because different staining protocols generally require adjacent serial sections rather than the same tissue section, and that Bland-Altman analysis was therefore not performed. As a partial substitute, we now specify that image-level mean CSA comparisons and animal-level mean and median CSA measurements were used as complementary summary-level assessments of morphometric consistency and biological differences, respectively.

5. Moderate overclaims.

Replace phrases such as “decisively prove,” “human-level accuracy,” and “completely preventing subjective human bias” with more scientifically cautious phrasing such as “support,” “within the range of inter-observer variability,” and “reducing the risk of subjective bias.”

(Response)

We have revised the manuscript language accordingly throughout the Results and Discussion sections. Specifically, "achieved human-level accuracy" has been changed to "within the range of inter-observer variability" (Page 15, Lines 337-338); "ensuring highly accurate and unbiased quantification" has been changed to "reducing the risk of subjective bias" (Page 10, Line 228); "highly effective substitute" has been changed to "practical substitute" (Page 20, Line 449); "equivalent between H&E- and TB-stained sections" has been changed to "comparable between H&E- and TB-stained sections" (Page 20, Lines 450-451). We believe these revisions provide a more balanced interpretation of our findings and align the language more closely with the strength of the supporting evidence.

6. Confirm archive completeness.

Ensure the Zenodo record contains raw per-fiber data, per-animal summary tables, representative input images, generated masks, the Fiji/ImageJ macro, local Python script, parameter documentation, README, and license/access statement.

(Response)

We have revised the Zenodo archive (https://doi.org/10.5281/zenodo.21482173), which is publicly accessible. The archive now contains a single Excel file (ParkJH_PO_raw data.xlsx) that includes the raw data underlying all quantitative analyses presented in Figs. 2-6 and Fig. S1, including raw per-fiber CSA measurements, image-level measurements, and per-animal summary data. It also contains representative input images, generated segmentation masks, the custom Fiji/ImageJ macro, the local Python script, parameter documentation, and a README file. We have also added the direct Zenodo URL alongside the DOI in the Code and Data Availability section to facilitate reviewer and reader access.

7. Strengthen limitations.

Add or refine limitations related to the small number of animals in the injury model, stain and scanner variability, possible performance differences in severe pathology/regenerating tissue, reliance on a hosted web service, and limited generalizability beyond mouse soleus sections.

(Response)

In response to the Reviewer’s comment, we have added a dedicated Limitations paragraph at the Discussion (Pages 21-22, Lines 478-501). Specifically, we discussed the limited number of animals evaluated in the denervation injury model (sham, n=3; SCI, n=4), the use of a single slide scanner, staining protocol, and mouse strain for validation, the lack of specific evaluation in severely dystrophic, necrotic, or actively regenerating muscle, the limitation of image- and animal-level rather than direct fiber-level cross-stain and cross-modality validation, and the current evaluation being limited to mouse soleus muscle. In addition, we acknowledge that the primary workflow relies on a publicly hosted web interface, although a local Python implementation is also provided as an alternative. These revisions provide a more balanced discussion of the limitations and potential applicability of the proposed workflow.

Attachment Submitted filename: Authors Responses to Reviewers Comments_revision2.docx

# Peer review: Decision Letter 2

**DOI:** 10.1371/journal.pone.0358476.r005

**Authors:** Masoud Rahmati

1 Sep 2026

Web-based Cellpose‑SAM segmentation enables quantification of cross-sectional area in formalin-fixed paraffin-embedded skeletal muscle sections stained with hematoxylin and eosin

PONE-D-25-67815R2

Dear Dr. KIM,

We’re pleased to inform you that your manuscript has been judged scientifically suitable for publication and will be formally accepted for publication once it meets all outstanding technical requirements.

Within one week, you’ll receive an e-mail detailing the required amendments. When these have been addressed, you’ll receive a formal acceptance letter and your manuscript will be scheduled for publication.

An invoice will be generated when your article is formally accepted. Please note, if your institution has a publishing partnership with PLOS and your article meets the relevant criteria, all or part of your publication costs will be covered. Please make sure your user information is up-to-date by logging into Editorial Manager at Editorial Manager® and clicking the ‘Update My Information' link at the top of the page. For questions related to billing, please contact billing support.

If your institution or institutions have a press office, please notify them about your upcoming paper to help maximize its impact. If they’ll be preparing press materials, please inform our press team as soon as possible -- no later than 48 hours after receiving the formal acceptance. Your manuscript will remain under strict press embargo until 2 pm Eastern Time on the date of publication. For more information, please contact onepress@plos.org.

Kind regards,

Masoud Rahmati

Academic Editor

PLOS One

Additional Editor Comments (optional):

Reviewers' comments:

Reviewer's Responses to Questions

**Comments to the Author**

Reviewer #1: All comments have been addressed

**********

2. Is the manuscript technically sound, and do the data support the conclusions?

Reviewer #1: Yes

**********

3. Has the statistical analysis been performed appropriately and rigorously?

Reviewer #1: Yes

**********

4. Have the authors made all data underlying the findings in their manuscript fully available?

The PLOS Data policy

Reviewer #1: Yes

**********

5. Is the manuscript presented in an intelligible fashion and written in standard English?

Reviewer #1: Yes

**********

Reviewer #1: Thank you for the careful and constructive revision. The revised manuscript is substantially improved. In particular, the clarified unit of inference, descriptive framing of pooled fiber-level KS distributions, added Dice/IoU summary metrics, expanded data/code availability, and strengthened limitations have addressed the major concerns from the previous review. I have only minor remaining suggestions before acceptance:

• Revise Figure 6 legend: replace “pooled mean CSA” and “pooled median CSA” with “group comparison of per-animal mean CSA” and “group comparison of per-animal median CSA.”

• Where KS tests are mentioned in figure legends, explicitly state “descriptive only” or remove wording that implies formal biological inference from pooled fibers.

• Soften “interchangeable” and “confirm” when referring to H&E and TB performance; use “comparable under the conditions tested.”

• Add “mouse soleus” or “mouse soleus sections” to the abstract conclusion to avoid implying universal applicability to other muscles, species, or clinical human archives.

• Check the Cellpose-SAM citation trail: cite the exact Cellpose-SAM paper/software/version used, in addition to Cellpose and SAM if appropriate.

• Correct incomplete reference formatting, especially reference 11, and add missing DOI/PMID where available.

These revisions are minor and should be straightforward to address. I consider the manuscript suitable for publication after these final corrections.

**********

what does this mean?). If published, this will include your full peer review and any attached files.

If you choose “no”, your identity will remain anonymous but your review may still be made public.

**Do you want your identity to be public for this peer review?**  For information about this choice, including consent withdrawal, please see our Privacy Policy

Reviewer #1: No

**********

# Editor report: Acceptance letter

**DOI:** 10.1371/journal.pone.0358476.r006

**Authors:** Masoud Rahmati

PONE-D-25-67815R2

PLOS One

Dear Dr. KIM,

I'm pleased to inform you that your manuscript has been deemed suitable for publication in PLOS One. Congratulations! Your manuscript is now being handed over to our production team.

At this stage, our production department will prepare your paper for publication. This includes ensuring the following:

* All references, tables, and figures are properly cited

* All relevant supporting information is included in the manuscript submission,

* There are no issues that prevent the paper from being properly typeset

You will receive further instructions from the production team, including instructions on how to review your proof when it is ready. Please keep in mind that we are working through a large volume of accepted articles, so please give us a few days to review your paper and let you know the next and final steps.

Lastly, if your institution or institutions have a press office, please let them know about your upcoming paper now to help maximize its impact. If they'll be preparing press materials, please inform our press team within the next 48 hours. Your manuscript will remain under strict press embargo until 2 pm Eastern Time on the date of publication. For more information, please contact onepress@plos.org.

You will receive an invoice from PLOS for your publication fee after your manuscript has reached the completed accept phase. If you receive an email requesting payment before acceptance or for any other service, this may be a phishing scheme. Learn how to identify phishing emails and protect your accounts at https://explore.plos.org/phishing.

If we can help with anything else, please email us at customercare@plos.org.

Thank you for submitting your work to PLOS One and supporting open access.

Kind regards,

PLOS One Editorial Office Staff

on behalf of

Dr. Masoud Rahmati

Academic Editor

PLOS One
